# Pharmacological targeting of kidney fibrosis: druggable mechanisms, translational models, and emerging antifibrotic therapies

**DOI:** 10.1093/ckj/sfag275

**Published:** 2026-08-12

**Authors:** Peijian Chen, Siyu Xie, Minglu Ding, Yanhui Chu, Jingru Wang

**Affiliations:** College of Life Sciences, Mudanjiang Medical University, Mudanjiang, Heilongjiang, PR China; Heilongjiang Province Key Laboratory for Anti-fibrosis Biotherapy, Mudanjiang Medical University, Mudanjiang, Heilongjiang, PR China; College of Life Sciences, Mudanjiang Medical University, Mudanjiang, Heilongjiang, PR China; School of Graduate Studies, Mudanjiang Medical University, Mudanjiang, Heilongjiang, PR China; College of Life Sciences, Mudanjiang Medical University, Mudanjiang, Heilongjiang, PR China; Heilongjiang Province Key Laboratory for Anti-fibrosis Biotherapy, Mudanjiang Medical University, Mudanjiang, Heilongjiang, PR China; College of Life Sciences, Mudanjiang Medical University, Mudanjiang, Heilongjiang, PR China; Heilongjiang Province Key Laboratory for Anti-fibrosis Biotherapy, Mudanjiang Medical University, Mudanjiang, Heilongjiang, PR China

**Keywords:** CKD, drug repurposing, immunometabolism, kidney fibrosis, precision medicine

## Abstract

Kidney fibrosis is the final common pathological pathway through which chronic kidney disease progresses to end-stage kidney disease, yet therapies designed specifically to interrupt the core fibrotic process in the kidney are still lacking. This unmet need reflects the biological heterogeneity of kidney fibrosis, the context-dependent interplay among inflammatory, metabolic and mechanical signals, and the limited translational value of many conventional preclinical models. The mechanistic landscape has also broadened considerably beyond canonical transforming growth factor-β signaling, now encompassing immune–stromal crosstalk, metabolic rewiring, mechanotransduction, epigenetic reprogramming, and extracellular vesicle-mediated communication. These developments have brought several druggable nodes into view and may support more selective and durable antifibrotic interventions. At the same time, translational platforms including artificial intelligence-assisted in silico screening, patient-derived kidney organoids, bioengineered tissue systems, and refined animal models are changing how targets are discovered and pharmacologically validated. In this review, we examine emerging mechanisms that govern kidney fibrogenesis, with emphasis on therapeutic tractability, and consider how experimental models can improve target prioritization and drug development. We also summarize repurposed drugs, pathway-targeted agents, receptor-directed strategies, and cell-based approaches under preclinical or clinical evaluation. We close by discussing key barriers to clinical translation, including disease heterogeneity, inadequate biomarkers, and the need to balance antifibrotic efficacy with renal safety. A pharmacology driven framework that links mechanism, model, and patient stratification could help accelerate precision antifibrotic therapy for kidney disease.

## INTRODUCTION

Kidney fibrosis is a major pathological process in the progression of chronic kidney disease (CKD) to end-stage kidney disease (ESKD) [1]. Although its clinical burden is substantial, therapeutic development remains difficult: no Food and Drug Administration (FDA)-approved treatment directly and specifically targets the core machinery of kidney fibrosis. Compared with antifibrotic progress in the heart, liver, and lung, the translational gap in nephrology is especially evident, reflecting barriers that still separate mechanistic discovery from clinical application. This gap makes precision medicine in kidney fibrosis an urgent priority. Several problems help explain the challenge: the absence of a unified classification framework that integrates distinct etiologies and molecular subtypes; the shortage of sensitive and specific biomarkers for dynamic monitoring and risk stratification; and the limited ability of most preclinical models to reproduce the complex, multicause pathophysiology of human kidney fibrosis.

This landscape has started to shift. Systems biology and human-tissue-based modelling platforms, including kidney organoids and organ-on-chip systems [1], are reshaping the study of fibrosis at molecular, cellular, and tissue levels. Evidence increasingly indicates that kidney fibrosis emerges from the convergence of systemic inflammation, metabolic stress, and mechanical loading, which together form a complex regulatory network [2, 3]. To make this complexity tractable, this review groups representative disease contexts according to dominant upstream biological drivers and uses an analytical framework that connects molecular mechanisms with clinical phenotypes.

Technological progress is also changing drug discovery. Stem-cell-derived kidney organoids and engineered tissue systems now enable more predictive, mechanism-guided screening of antifibrotic compounds [1]. These platforms have helped repurpose agents first evaluated in early clinical trials and have made it easier to translate mechanistic insights involving transforming growth factor (TGF)-β signaling, renin–angiotensin–aldosterone system (RAAS) activation, immune–fibroblast interactions, and metabolic remodeling into targeted antifibrotic strategies [4–7].

The review is structured around three themes. We first examine the core mechanisms of kidney fibrosis and the system-level logic that links them. We then discuss the major translational model systems used to study fibrotic remodeling, including in silico, *in vitro*, and *in vivo* platforms. Finally, we summarize antifibrotic agents now in preclinical development or clinical testing, before considering the main challenges and future directions for precision-guided antifibrotic therapy in kidney disease.

## KIDNEY FIBROSIS IN CONTEXT: DISTINCTIVE FEATURES ACROSS ORGAN SYSTEMS

Fibrosis represents an exaggerated repair response across many organs, including the kidney, heart, liver, and lung; nevertheless, both the mechanisms involved and their pathological consequences are strongly shaped by the organ in which fibrosis occurs [8] (Table 1). Cardiac fibrosis primarily affects the myocardial interstitium and leads to electromechanical dysfunction, whereas kidney fibrosis often begins with dysfunction or loss of tubular epithelial cells or podocytes. This injury activates resident interstitial fibroblasts and pericytes, which represent the principal source of extracellular matrix (ECM)-secreting myofibroblasts and constitute a key initiating event in renal fibrogenesis. Injured tubular epithelial cells primarily acquire a partial epithelial–mesenchymal transition (EMT)-associated secretory phenotype that promotes paracrine activation of neighboring fibroblasts; however, accumulating lineage-tracing evidence demonstrates that these cells do not directly transdifferentiate into ECM-secreting interstitial myofibroblasts *in vivo* [9].

**Table 1:** Comparison of kidney fibrosis with fibrosis in other organs.

Feature	Kidney	Heart	Liver	Lung
Cellular sources of fibrosis	Resident fibroblasts; pericytes; tubular epithelial cells acquiring EMT-associated secretory phenotype (paracrine effects)	Resident fibroblasts; epicardium-derived cells; endothelial cells acquiring endothelial-to-mesenchymal transition (EndoMT)-associated secretory phenotype (paracrine effects)	Hepatic stellate cells; portal fibroblasts	Resident stromal cells; alveolar epithelial cells acquiring EMT-associated secretory phenotype (*in vivo* full transdifferentiation remains debated)
Immune–fibroblast crosstalk	TGF-β, interleukin (IL)-1β, TNF-α; monocyte/macrophage (M1/M2/M2c)–fibroblast interactions	IL-1β axis; SiglecF+ macrophage-driven fibroblast activation; CCL17–CCR4 axis	Kupffer cell-hepatic stellate cell signaling; TNF-α/IL-6-mediated inflammation–fibrosis crosstalk	SiglecF+ macrophages; IL-1β and TGF-β-driven immune-fibroblast signaling
Metabolic regulation	Mitochondrial dysfunction, lipotoxicity, glucose metabolic reprogramming; dysregulated sirtuin/AMPK signaling	Mitochondrial dysfunction; impaired fatty acid oxidation	Disordered bile acid metabolism; farnesoid X receptor (FXR)/peroxisome proliferator-activated receptor (PPAR) pathways; glucose/lipid toxicity	Oxidative stress; imbalance in nuclear receptor signaling
Translational therapeutic status	No FDA-approved kidney-specific antifibrotic drug; SGLT2 inhibitors and MRAs show antifibrotic effects in CKD	No FDA-approved organ-specific antifibrotic drug; pirfenidone and nintedanib remain in clinical evaluation	Obeticholic acid approved for fibrosing cholangiopathy; no approved drug for non-alcoholic steatohepatitis (NASH) fibrosis	Pirfenidone and nintedanib approved for idiopathic pulmonary fibrosis

EMT/EndoMT in this table describes partial phenotypic transformation accompanied by secretory changes. Accumulating lineage-tracing evidence indicates that epithelial or endothelial cells do not fully transdifferentiate into interstitial myofibroblasts *in vivo*.

Although aberrant immune–fibroblast crosstalk is a common feature of fibrosis across organs, the participating immune populations are organ specific. In the kidney, imbalance in M1 (classically activated) and M2 (alternatively activated) macrophage polarization [10] and dendritic-cell-mediated adaptive immune responses are key regulators [11]; this differs substantially from the regulatory networks dominated by SiglecF+ macrophages in the lung [12] and Kupffer cells in the liver [13]. The kidney also appears to harbor a distinctive profibrotic role for M2c macrophages, which promote myofibroblast activation by secreting TGF-β [10], with Janus kinase 2 (JAK2)/signal transducer and activator of transcription 3 (STAT3) signaling serving as a central regulator of their polarization. This mechanism has not yet been clearly established in fibrosis of other organs. At the metabolic level, kidney fibrosis is likewise organ specific, being closely linked to mitochondrial dysfunction, disordered lipid metabolism, hypoxia-inducible factor signaling, and vitamin D metabolic abnormalities [14–17]. These features sharply contrast with nuclear receptor signaling in pulmonary fibrosis and bile acid metabolism in liver fibrosis. Consistent with these differences, drugs such as pirfenidone and nintedanib, which have shown efficacy in preclinical or selected clinical settings of liver or lung fibrosis, have yielded inconsistent or limited benefit in kidney fibrosis. Pirfenidone has shown only modest potential to slow glomerular filtration rate decline in small studies [18], whereas nintedanib has thus far been documented mainly as safe in patients with kidney disease complicated by chronic pulmonary fibrosis, with kidney-specific efficacy still unproven [19]. These observations point to the need for developing strategies grounded in kidney-specific biology.

### Druggable mechanisms in kidney fibrosis

Kidney fibrosis has often been understood through canonical pathways such as TGF-β, angiotensin II (Ang II), and Wnt/β-catenin, all of which promote fibroblast activation [20]. During CKD progression, canonical pathways such as TGF-β, Ang II, and Wnt/β-catenin converge on quiescent resident fibroblasts and pericytes, driving their differentiation into ECM-secreting myofibroblasts and thereby promoting renal scarring and nephron loss. These same cues also induce injured tubular epithelial cells to adopt a partial EMT-associated secretory phenotype that amplifies fibroblast activation through paracrine signaling; lineage-tracing studies confirm that such epithelial cells do not directly transdifferentiate into interstitial myofibroblasts *in vivo* [9]. This framework remains foundational, but it is no longer sufficient on its own. Canonical fibrotic pathways are tightly linked with immune-inflammatory signaling, metabolic reprogramming, and mechanosensing networks [2, 3], so fibrotic signaling should be viewed as a context-dependent system shaped by cell type and disease etiology. This broader view offers a higher-resolution account of pathogenesis and points to new therapeutic opportunities. Targeting convergence points, such as inflammation-driven epigenetic memory or mechanosensitive regulators, may overcome some limitations of simple upstream blockade and yield more durable and selective antifibrotic effects [21]. Nontraditional targets, including alternative paracrine circuits, further widen the strategic landscape for precision therapy in kidney fibrosis.

### Inflammatory and immune signaling

**Figure 1:**
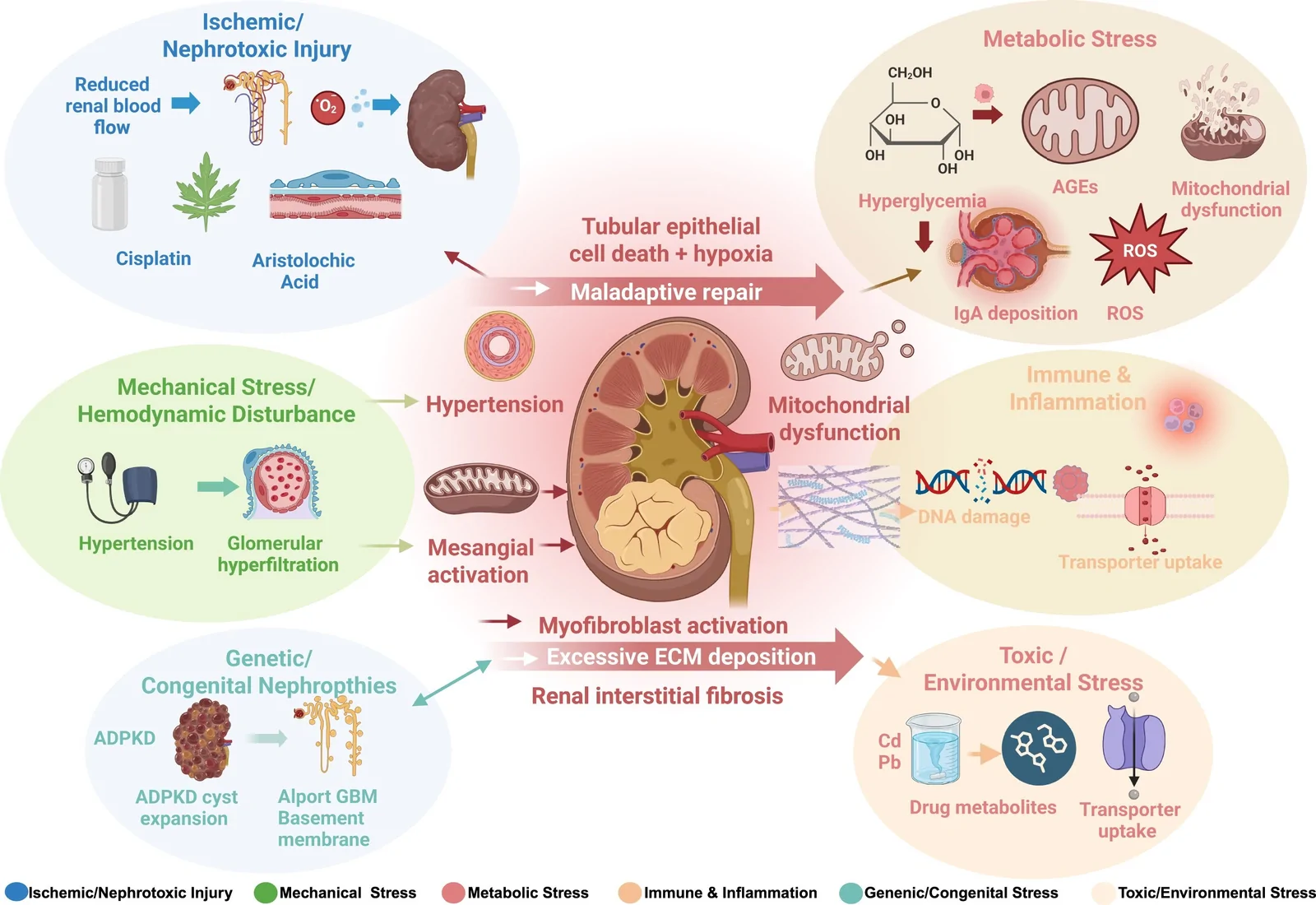
Mechanistic contexts of kidney fibrosis across disease states. This schematic summarizes six key pathological stimuli that drive kidney fibrosis, including ischemic/nephrotoxic injury, mechanical stress, metabolic stress, immunity and inflammation, toxic/environmental stress, and genetic/congenital disease. These processes collectively promote maladaptive repair and renal interstitial fibrosis. Created with BioRender.

Inflammation and immunity are key drivers of kidney fibrosis. Sustained immune–stromal interactions promote fibroblast activation and ECM remodeling (Fig. 1), and recent studies have identified several immunoregulatory targets that may support more precise interventions.

## MECHANISTIC CONTEXTS OF KIDNEY FIBROSIS: REPRESENTATIVE DISEASE ASSOCIATIONS

Kidney fibrosis is the common final pathway of CKD progression, but its pattern, pace, and treatment response depend heavily on disease etiology [2, 22]. The sections below outline selected biological mechanisms associated with specific disease settings, as illustrated in Fig. 1.

### Ischemic and nephrotoxic injury

Reduced renal blood flow and nephrotoxic agents such as cisplatin or aristolochic acid can induce tubular epithelial cell death and hypoxia [23], initiating maladaptive repair programs [24, 25]. During the transition from acute kidney injury (AKI) to CKD, ongoing cell death promotes myofibroblast activation and ECM deposition through TGF-β signaling [26], inflammatory responses [27], and endothelial dysfunction [28].

### Mechanical stress and hemodynamic disturbance

Systemic or intrarenal hemodynamic changes, including hypertension and intraglomerular hypertension, expose renal parenchymal cells to chronic biomechanical stress and promote fibrosis. In diabetic kidney disease, glomerular hyperfiltration and elevated intratubular pressure act as major profibrotic forces [29]. Hyperglycemia and local renin–angiotensin system activation may amplify mechanical stimulation of mesangial cells and induce secretion of profibrotic mediators. In obesity-related kidney disease, hyperfiltration-related mechanical stress is intensified by ectopic lipid accumulation and protein overload in the kidney; lipid-induced insulin resistance further worsens maladaptive responses to mechanical stress and promotes fibroblast activation and interstitial fibrosis through oxidative-stress pathways [30].

### Metabolic stress and immunometabolic rewiring

Metabolic derangement promotes kidney fibrosis through oxidative stress, mitochondrial dysfunction, and lipotoxicity. In diabetic kidney disease, persistent hyperglycemia and advanced glycation end products activate profibrotic pathways such as TGF-β and PI3K/Akt [31, 32]. Kidney injury associated with metabolic syndrome is also closely related to insulin-resistance-driven, low-grade chronic inflammation; cytokines such as interleukin-6 can promote excessive ECM deposition by driving fibroblast-to-myofibroblast transition [33].

### Immunity and inflammation

Chronic autoimmunity and inflammation drive several fibrotic kidney diseases [34]. In lupus nephritis, immune-complex deposition and complement activation provoke intense inflammation, ultimately leading to glomerulosclerosis and interstitial fibrosis [35]. In immunoglobulin A (IgA) nephropathy, mesangial deposition of IgA immune complexes activates mesangial cells and promotes fibrosis through TGF-β secretion [36]. In antineutrophil cytoplasmic antibodies (ANCA)-associated vasculitis, neutrophil infiltration injures the vascular wall and further contributes to tubulointerstitial fibrogenesis [37].

### Toxic injury and environmental stress

Because the kidney is a major excretory organ, it is particularly vulnerable to environmental toxicants and drug-induced injury. In cisplatin- or aristolochic-acid-induced nephropathy, the drug or its metabolites damage tubular epithelial DNA and provoke oxidative stress, initiating chronic fibrotic programs [23, 38]. Cisplatin uptake depends on organic cation transporters expressed on the basolateral membrane of proximal tubular cells [39, 40]; after internalization, cisplatin forms DNA adducts, blocks DNA replication, and ultimately causes irreversible cell death followed by a fibrotic cascade [38]. Heavy-metal exposure, including cadmium or lead, can also accelerate fibrosis by suppressing antioxidant enzyme activity and inducing mitochondrial dysfunction [41, 42].

### Genetic and congenital kidney disease

Genetic mutations that disrupt renal structure or function can drive fibrosis directly or indirectly. In autosomal dominant polycystic kidney disease (ADPKD), cyst formation and expansion mechanically compress adjacent healthy tissue and cause local ischemia, promoting fibrosis [43]. In Alport syndrome, mutations in collagen genes of the glomerular basement membrane impair the filtration barrier and lead to progressive glomerulosclerosis and interstitial fibrosis through repeated cycles of epithelial injury and repair [44]. In the phase 3 FALCON trial (NCT03918447), the Nrf2 activator bardoxolone methyl was evaluated for its ability to slow estimated glomerular filtration rate (eGFR) decline in ADPKD through suppression of cyst-associated inflammation and interstitial fibrosis; however, the trial was terminated after failing to meet its primary efficacy endpoints, underscoring the challenges of translating preclinical antifibrotic mechanisms into durable clinical benefit in complex genetic nephropathies.

Persistent inflammatory signaling strongly shapes fibrotic remodeling, with cytokine signaling occupying a central position in this process. Tumor necrosis factor (TNF)-α can initiate fibrotic cascades by inducing metabolic reprogramming and senescence in tubular epithelial cells, promoting sustained monocyte/macrophage infiltration and activating local TGF-β1 signaling to stimulate fibroblasts [45]. For this reason, it is considered a major driver of fibrosis in diabetic kidney disease. Recent work also suggests that TNF-α can promote long-range myofibroblast activation by regulating the release of EMT-associated exosomes from tubular epithelial cells. Multi-omic and spatial analyses of kidney biopsies from patients with diabetic kidney disease and IgA nephropathy further indicate that TNF-α-mediated tubular–macrophage crosstalk links inflammation with fibrotic remodeling, driving expansion of alpha–smooth muscle actin (α-SMA)/platelet–derived growth factor receptor beta (PDGFRβ)+ double-positive myofibroblasts with enhanced collagen-synthetic capacity [46–48]. These observations position TNF-α as a mechanism-based antifibrotic target at the intersection of immune signaling and fibroblast activation.

Targeting the nodal points that maintain fibroblast activation is therefore a practical route for mechanism-guided antifibrotic therapy. Epigenetic regulation is one such node. In unilateral ureteral obstruction (UUO)-induced chronic injury, histone deacetylase (HDAC)-mediated chromatin remodeling in tubular epithelial cells and mothers against decapentaplegic homolog (Smad)3-dependent enhancer activation in fibroblasts help maintain persistent expression of profibrotic genes. This provides one molecular explanation for disease persistence. HDAC9 contributes to renal fibrosis by inducing G2/M cell-cycle arrest in tubular epithelial cells [49], whereas HDAC3 activates profibrotic pathways by suppressing Klotho transcription [50]. These findings suggest that inflammation-induced transcriptional memory may be reversible through epigenetic intervention, placing HDACs among candidate antifibrotic targets that could be combined with RAAS blockers or sodium–glucose cotransporter 2 (SGLT2) inhibitors in precision therapeutic strategies. The C–C motif chemokine ligand (CCL)2–C–C motif chemokine receptor (CCR)2 chemokine axis is another important pathway. It promotes recruitment and activation of inflammatory M1 macrophages while suppressing transition toward reparative M2 phenotypes [51]. By pushing macrophages toward a proinflammatory state, this axis amplifies the inflammatory-fibrotic cascade in the renal interstitium. Selective CCL2 blockade restores homeostatic phagocytic function, reduces interstitial collagen deposition in UUO mice, and improves renal function indices such as serum creatinine, supporting macrophage phenotype modulation as a translational antifibrotic strategy [52].

Metabolic and endocrine mediators add further complexity to inflammatory networks in kidney fibrosis. Fibroblast growth factor 23 (FGF23) is a notable example. In a clinical study of 128 patients with stage 3–5 CKD, elevated circulating FGF23 levels were significantly associated with lower glomerular filtration rate, greater tubular atrophy, and more extensive interstitial collagen deposition, identifying FGF23 as an independent risk factor for progressive interstitial fibrosis and glomerulosclerosis [53]. These data support FGF23 as a clinically relevant profibrotic driver in CKD, and therapies targeting FGF23 signaling, including fibroblast growth factor receptor (FGFR) inhibitors, may have future antifibrotic potential [54]. In contrast, α-Klotho, produced mainly by proximal tubular cells, suppresses tubular injury and interstitial fibrosis by antagonizing Wnt/β-catenin and TGF-β1/Smad3 signaling. Because Wnt/β-catenin lies downstream of TGF-β/Smad and Klotho inhibits this pathway, Klotho downregulation promotes Wnt activation and worsens renal fibrosis [55, 56]. Through suppression of oxidative stress, cellular senescence, and fibroblast activation [53], α-Klotho represents a renal endocrine axis with plausible antifibrotic relevance, particularly in CKD and pregnancy-related acute kidney injury (p-AKI) fibrosis. Circulating Klotho deficiency in CKD is increasingly recognized as a systemic endocrine disturbance that extends beyond the kidney while further worsening the local fibrotic milieu [53, 55, 56]. Overall, metabolic-endocrine dysregulation is a therapeutically tractable component of kidney fibrosis in diabetic kidney disease, hypertensive nephrosclerosis, ischemia-reperfusion injury, and immune-complex-mediated glomerulonephritis. Current guidelines have already incorporated SGLT2 inhibitors and nonsteroidal mineralocorticoid receptor antagonists (MRAs) into core CKD management [57], while precision targeting of epigenetic circuits, chemokine axes, and metabolic-endocrine mediators could further expand combination therapy for fibrotic kidney disease [2].

## MECHANOTRANSDUCTION AND MATRIX SENSING

**Figure 2:**
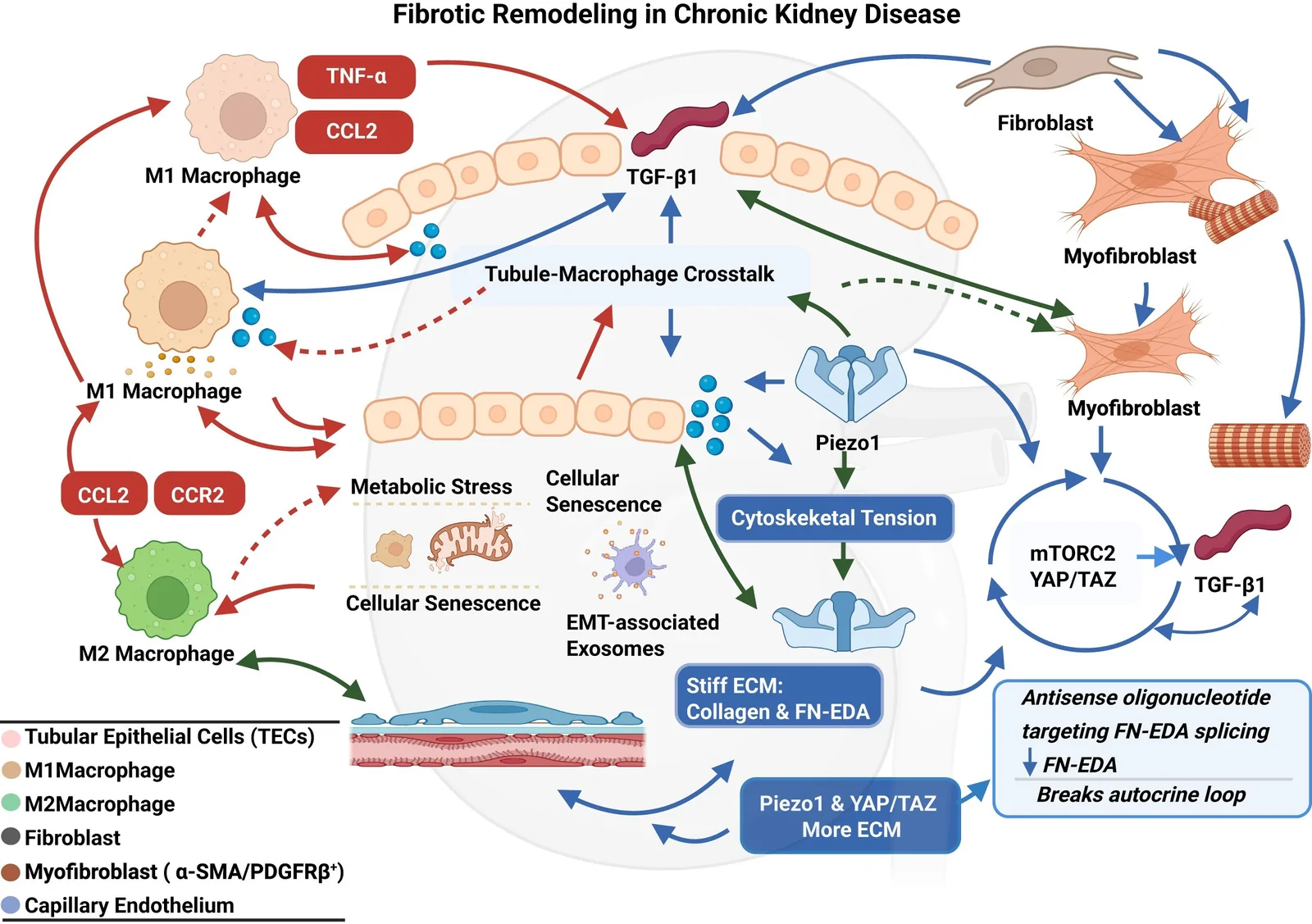
Dual axes of fibrotic regulation: immune crosstalk and mechanical feedback in kidney fibrosis. This schematic illustrates two interconnected pathways driving kidney fibrosis: immune-mediated crosstalk and mechanical feedback signaling. Immune signals and mechanical cues converge to promote myofibroblast activation and excessive ECM deposition. Created with BioRender.

Mechanical signaling is increasingly recognized as a major regulator of kidney fibrosis. During CKD progression, glomerular hypertension, elevated tubular luminal pressure, and tissue stiffening caused by interstitial ECM accumulation are converted into cellular responses by the mechanosensitive receptor PIEZO1. This process tightly couples matrix dynamics to dysfunction and activation of resdent renal cells, including fibroblasts, podocytes, and tubular epithelial cells [58–60]. A defining feature of kidney fibrosis is the biomechanical feedback loop created by sustained interactions between cells and the surrounding ECM (Fig. 2), where profibrotic and proinflammatory pathways are closely intertwined. Inflammatory mediators such as interleukin–1 beta (IL-1β) and TNF-α can regulate mechanosensitive pathways, including Hippo-Yes–associated protein (YAP)/transcriptional co–activator with PDZ–binding motif (TAZ), thereby linking inflammatory signaling to ECM remodeling during renal fibrogenesis [58, 60, 61]. This pattern resembles Piezo1-mediated interactions between mechanical and inflammatory pathways and is consistent with shared principles in fibrosis and inflammation. Stretch, fluid shear stress, and matrix stiffness activate renal cells through conserved mechanotransduction routes, influencing both the spatial pattern of fibrosis, such as focal scarring versus diffuse interstitial fibrosis, and its persistence [58, 59]. Paracrine mechanical signaling is particularly relevant: activated myofibroblasts stimulate neighboring tubular epithelial cells and quiescent fibroblasts through integrin αv/β5, DDR1, Piezo1, and focal adhesion complexes, promoting myocardin–related transcription factor (MRTF)-A/serum response factor (SRF)-driven transcriptional programs [62] and driving fibrosis independently of soluble factors [63, 64]. Together, these data support mechanoreceptors and cytoskeletal regulators as potential therapeutic targets in kidney fibrosis [65].

Within this framework, TGF-β1 and YAP/TAZ appear to cooperate in the stiffened renal interstitium and generate a self-amplifying mechanical-paracrine feedback loop. TGF-β1 activates YAP/TAZ through mTORC2 to drive fibroblast activation [66], while excessive YAP/TAZ activation reinforces TGF-β signaling and further aggravates myofibroblast differentiation and matrix deposition [67]. The convergence of mechanical and biochemical signaling is especially relevant in hyperfiltering, high-pressure nephron regions, suggesting that disrupting this synergy may be necessary to halt fibrotic progression. Glycogen synthase kinase (GSK)-3β has also emerged as an important mechanosensitive regulator in the kidney [68]. Current evidence indicates that it sustains fibroblast activation through the JNK/c-JUN pathway independently of canonical Smad3 signaling, offering an alternative therapeutic route when direct TGF-β targeting is insufficient.

Matrix-cell feedback also has a strong influence on fibroblast fate. The fibronectin splice variant FN-EDA accumulates in fibrotic kidneys, and antisense oligonucleotides that selectively inhibit FN-EDA splicing can reduce its production and interrupt autocrine profibrotic loops [69]. FN-EDA also regulates proinflammatory and profibrotic responses in a microenvironment-dependent manner, making this signaling network an attractive intervention point. Another important concept is mechanical memory: fibroblasts and tubular epithelial cells can retain a profibrotic phenotype after the initial mechanical stimulus has ceased, partly through epigenetic modifications. In tubular epithelial cells, METTL3-mediated m6A modification of Ena/VASP-like protein (EVL) has been identified as a core mechanism of this memory after matrix stiffness stimulation [70], allowing cells to continue moving toward a profibrotic state even after the mechanical cue is removed. This persistent imprint extends the therapeutic window beyond the acute injury stage and supports durable therapies aimed at reversing epigenetic reprogramming in fibrotic cells. Understanding how mechanical cues stabilize cell states may reveal ways to dismantle the self-sustaining circuitry of kidney fibrosis at its source.

## EMERGING MOLECULAR PATHWAYS AND THERAPEUTIC TRACTABILITY

Kidney fibrosis is increasingly not viewed as a process driven only by canonical cascades centered on TGF-β, Ang II, and ECM signaling. It is better understood as a system-level disease process shaped by modular, multilayered regulatory networks [22]. Recent studies have identified mechanisms involving metabolic reprogramming [71], epigenetic regulation [72], abnormal intercellular communication, and dysregulated transcriptional programs [22]. These pathways are highly cell-type specific and microenvironment dependent, and they intersect with inflammation, hypoxia, and mechanical tension to regulate fibroblast activation and matrix deposition. Rather than operating independently, they integrate with classical fibrotic pathways to shape fibroblast to myofibroblast transdifferentiation and secretory function in specific cellular and disease contexts.

Upstream drivers, downstream targets, and key effectors in kidney fibrosis span numerous axes, including intrarenal renin–angiotensin signaling (RAS-Ras/Raf-1/ERK1/2) [73], the metabolic reprogramming axis PC-SQOR/cGAS/STING-glycolysis [74], noncoding RNA regulatory networks such as lncRNA GAS5-miR-142-5p [75], paracrine and extracellular vesicle signaling such as ADSC-EVs/FOXS1/Wnt/β-catenin [76], cytokine pathways including TNC-TLR4-NF-κB-miR-155-5p [77], energy-sensing routes such as ACSS2-H3K9cr-IL-1β [78], transcriptional regulation including Wnt/β-catenin-DKK3-MMF [79], and cell cycle regulators such as p53-p21 [80]. Together, these mechanisms form a densely interconnected, multi-axis regulatory framework.

Most of these pathways have already been supported by preclinical models. Notably, interventions targeting TGF-β signaling and its upstream regulators, such as Ang II receptors, have already entered clinical testing in CKD. In contrast, the PC-SQOR/cGAS/STING-glycolysis axis represents a newer mechanistic theme that has been validated in tubular epithelial cell-specific knockout models, providing a new candidate target for fibrotic CKD. Overall, kidney fibrosis is shaped by both conserved core hubs and tissue-specific auxiliary regulators. Hubs such as TGF-β, the mechanochemical sensors YAP/TAZ, and the metabolic integrators pyruvate carboxylase (PC) and SQOR function as upstream nodes that connect mitochondrial injury, innate immune activation, and glycolytic reprogramming. In contrast, noncoding RNAs, extracellular vesicles, and epigenetic pathways more often act as downstream or parallel regulators that shape the phenotype and trajectory of fibrosis in disease-specific contexts such as diabetic kidney disease, ischemia-reperfusion injury, or obstructive nephropathy. Broadly active pathways, such as TGF-β/Smad3, are commonly engaged across fibrotic states, whereas more selective pathways such as PC-SQOR/cyclic GMP–AMP synthase (cGAS)/stimulator of interferon genes (STING)-glycolysis may offer disease-specific intervention opportunities. Defining the spatiotemporal activation of these pathways across tubular epithelial cells, interstitial fibroblasts, nephron compartments, and clinical phenotypes will be essential for identifying actionable targets and designing rational combination therapies. Because these mechanisms are dynamic and potentially reversible, and because they respond selectively to disease cues such as injury and inflammation, they are attractive for precision antifibrotic intervention.

## EXPERIMENTAL MODELS FOR PHARMACOLOGICAL DISCOVERY AND VALIDATION

**Figure 3:**
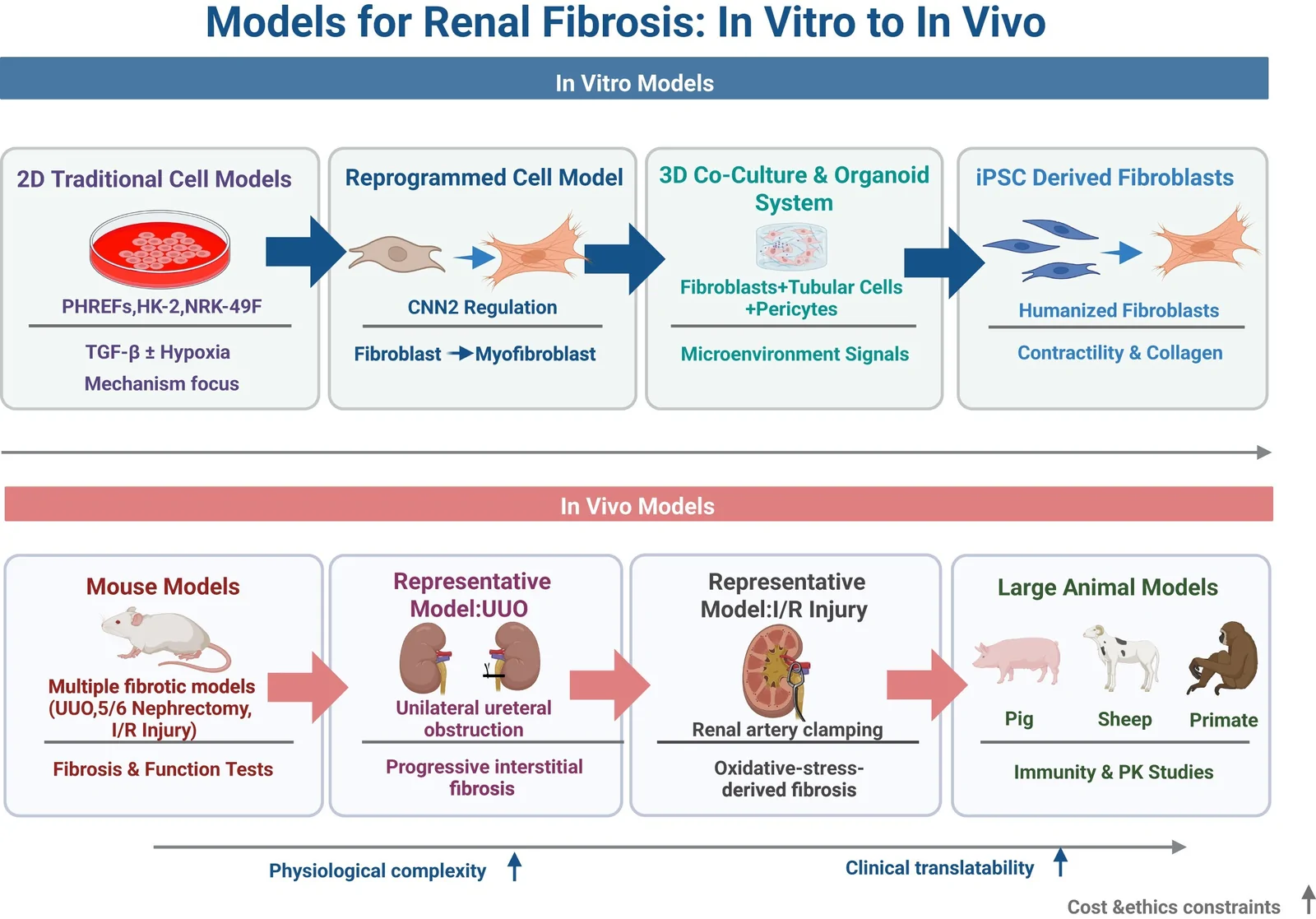
Experimental platforms for modeling kidney fibrosis from *in vitro* to *in vivo*. This schematic summarizes key experimental models used in kidney fibrosis research, spanning *in vitro* systems (including 2D culture, 3D co-culture/organoid platforms, and iPSC-derived models) and *in vivo* models. Created with BioRender.

Progress in antifibrotic therapy for CKD depends on experimental models that reproduce fibrotic remodeling across molecular, cellular, and tissue scales with sufficient fidelity. In recent years, integrated in silico, *in vitro*, and *in vivo* platforms have been developed to model key mechanisms of tubulointerstitial injury, stratify therapies across CKD subtypes, and accelerate translation of antifibrotic strategies. Together, these systems support mechanism-based target discovery, scalable drug screening, and individualized efficacy assessment, and are beginning to provide a foundation for precision-guided antifibrotic research and clinical application in kidney fibrosis (Fig. 3).

### In silico models: from target discovery to predictive pharmacology

Artificial intelligence (AI) and computational modeling now play a growing role in antifibrotic drug development and in the interpretation of the complex remodeling processes associated with kidney fibrosis. Current computational platforms integrate machine learning, molecular docking, and molecular dynamics simulation. In renal fibrosis associated with diabetic kidney disease, e.g. transcriptomics-guided machine learning combined with docking and dynamics simulation identified FPR1, TAS2R5, and LRP2BP as key targets mediating the antifibrotic effects of valsartan [81], showing the value of integrated computational frameworks for target identification and treatment prediction. Molecular docking has also helped screen targets for other compounds. Diosmin, for instance, was predicted to bind fatty-acid-binding protein 4 (FABP4), and subsequent work confirmed that it suppresses fibrosis by inhibiting the nuclear factor kappa–light–chain–enhancer of activated B cells (NF-κB)/FABP4 axis [82]. Mechanistic analyses and validation in UUO mice further showed that modulation of FABP4-related pathways attenuates tubular injury and ECM accumulation. Three-dimensional organoid models based on renal anatomy have also suggested that extracellular vesicles secreted by tubular epithelial cells can reshape the interstitial microenvironment through microRNA (miRNA) transfer and may even reverse early ECM deposition, a result consistent with computational predictions and preclinical animal studies [83]. In pathology, machine-learning-based models for evaluating interstitial fibrosis in kidney biopsies have achieved 0.90 agreement with expert pathologists and correlate strongly with renal functional indices, supporting the reliability of AI in fibrosis-related prediction [84]. Although current AI models remain constrained by the availability of high-quality, kidney fibrosis-specific training datasets, early applications in diabetic kidney disease suggest value in distinguishing SGLT2 inhibitor-sensitive from resistant subtypes and guiding individualized therapy. Even without full molecular-resolution validation, these tools show promise for accelerating drug discovery, strengthening biomarker development, and advancing precision treatment in kidney fibrosis.

### *In vitro* systems: from 2D culture to patient-derived kidney organoids

*In vitro* models are useful for dissecting the molecular basis of kidney fibrosis and screening antifibrotic compounds because they allow controlled fibroblast activation, ECM deposition, and high-throughput assessment of drug responses. These platforms have moved from simple two-dimensional cultures to complex three-dimensional tissue constructs and, more recently, patient-derived kidney organoids. Three-dimensional kidney organoids represent an advanced *in vitro* platform that bridges traditional cell culture and animal models (Fig. 3). They now increasingly recapitulate structural remodeling, altered mechanical stress, and physiological dysfunction during renal fibrogenesis, and can reproduce spatiotemporal disease features and tubular–interstitial crosstalk (Fig. 3). Traditional 2D systems based on primary human renal fibroblasts, immortalized renal epithelial lines such as HK-2, or rodent fibroblasts such as NRK-49F are usually induced with profibrotic stimuli including TGF-β and hypoxia [83]. Although these systems remain useful for mechanistic analysis, they often miss the disease-relevant phenotypic transitions seen in CKD. Models built around calponin 2-related pathways can better mimic injury-induced cellular reprogramming and key features of fibroblast-to-myofibroblast conversion [85]. Co-culture systems improve physiological relevance further by integrating fibroblasts, pericytes, and tubular epithelial cells into compartmentalized three-dimensional models that better reproduce the renal pathological microenvironment and allow more accurate analysis of non-cell-autonomous paracrine regulation [86]. Human induced pluripotent stem-cell (iPSC)-derived renal fibroblast models provide a closer approximation to human pathology. When incorporated into organoid systems, they allow quantitative assessment of contractility, expression of fibrosis-associated genes such as Col1a1 and α-SMA, and Smad3-mediated epigenetic remodeling using collagen gel contraction assays, quantitative reverse transcription polymerase chain reaction (qRT-PCR), and chromatin immunoprecipitation quantitative polymerase chain reaction (ChIP-qPCR)[87]. Three-dimensional kidney organoids further improve clinical relevance by mimicking multicellular architecture and dynamic disease progression. Single cell and organoid engineering studies show that iPSC-derived kidney organoids exposed to ischemia-reperfusion injury or TGF-β1 can reproduce key pathological features of ischemia-associated renal fibrosis, including excessive collagen deposition, disrupted calcium homeostasis in tubular epithelial cells, and sustained activation of TGF-β/Smad2/3 signaling. They can also model the heterogeneity of peritubular fibroblast populations, now recognized as part of fibrotic progression. A 2024 study using three-dimensional bioprinted tubulointerstitial organoids showed that this humanized platform can reproduce fibrotic cascades, reveal the spatiotemporal association between endothelial injury and fibrosis *in vitro*, and support pharmacological testing with pirfenidone [88]. Organoids still have limitations, including incomplete functional maturation, structural simplification, lack of vascularization, and potential immune incompatibility. These constraints are driving efforts to combine organoid systems with *in vivo* animal models to interrogate disease mechanisms and evaluate drug efficacy more comprehensively.

### *In vivo* models: from small animals to large animals

*In vivo* models are essential for simulating the pathological course of kidney fibrosis and for evaluating antifibrotic therapies, with translational relevance increasing from mouse models to large-animal systems (Fig. 3). In mice, multiple injury paradigms such as UUO and 5/6 nephrectomy model obstructive nephropathy and CKD-related fibrosis [22, 89]. In contrast, folic-acid-induced injury models reproduce the pathological transition from AKI to chronic fibrosis through calcium oxalate crystal deposition and tubular metabolic disruption, making them particularly suitable for studying this transition [90]. Ischemia-reperfusion models accurately capture inflammation-driven interstitial fibrosis after renal ischemia [91]. These systems reproduce hallmark features including epithelial plasticity associated with partial EMT-like molecular changes, fibroblast activation and proliferation [92], excessive collagen deposition [93], and impaired glomerular filtration, and have been widely used to test agents such as pirfenidone, curcumin, and small-molecule inhibitors targeting the TGF-β/Smad pathway [94]. High-fat diet plus streptozotocin models combine metabolic dysregulation with hyperglycemic stress and recapitulate key features of fibrosis in metabolic kidney disease [95]. Transgenic and knockout models, such as TGF-β1 overexpression [96] or miR-29b deficiency [97], help reveal context-specific mechanisms of fibrosis. Other models, including MCU overexpression [98] or TP53 deletion, are also informative [99] even when fibrosis is not the dominant phenotype, because they illuminate pathways involving mitochondrial calcium handling, tubular senescence, and age-related remodeling that contribute to fibrogenesis. These models are valuable not because they universally induce fibrosis, but because they isolate critical regulatory nodes and identify the cell subsets that drive disease progression. Large-animal models add physiological relevance and allow translational assessment of immune responses, toxicity, and pharmacokinetics. In pigs, high-fat diet-induced fibrosis in Bama miniature pig models human hypertensive renal fibrosis, and renal denervation studies have further demonstrated translational utility [100]. In sheep, postcardiopulmonary bypass renal hypoxia induces sustained hypoxic injury and serves as a useful model for surgery-related fibrosis [101]. In nonhuman primates, UUO in cynomolgus monkeys reproducibly mirrors human obstructive kidney fibrosis with activation of TGF-β1/Smad signaling [102], offering an important platform for preclinical drug validation. Despite these advantages, large-animal models remain limited by cost, restricted genetic tools, and animal-welfare concerns. Taken together, *in vivo* models from transgenic mice to large-animal systems bridge basic mechanistic insights and preclinical validation by reproducing the key molecular and structural features of kidney fibrosis in disease-relevant contexts.

## CURRENT AND EMERGING ANTIFIBROTIC THERAPIES

Building on these model systems, preclinical studies have tested a widening range of antifibrotic therapies, including repurposed drugs and newer targeted strategies. These approaches now represent some of the most active routes for mechanism-based and translational intervention in kidney fibrosis.

### Repurposed and investigational drugs for kidney fibrosis

**Figure 4:**
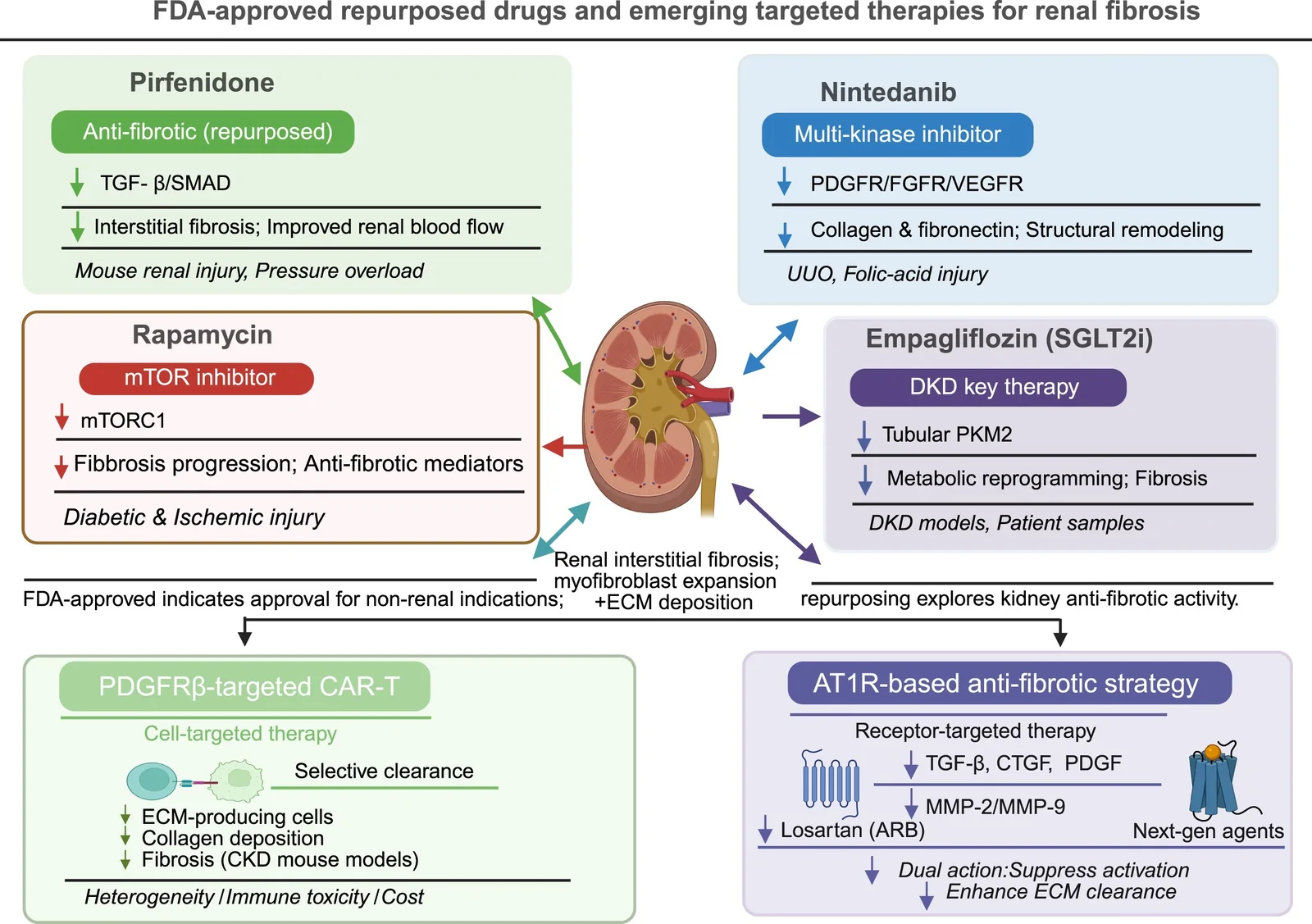
FDA-approved drugs with potential antifibrotic activity in kidney fibrosis. This schematic summarizes FDA-approved drugs being repurposed for kidney fibrosis, targeting key profibrotic signaling pathways. These agents act on distinct mechanisms to reduce ECM deposition and slow fibrotic progression. Created with BioRender.

A growing number of drugs approved by the FDA for nonrenal indications are being examined for antifibrotic activity in kidney disease. Although kidney fibrosis has a distinct molecular landscape, it shares core regulatory pathways with fibrosis in the heart, liver, and lung, including TGF-β signaling and myofibroblast activation [103]. This cross-organ overlap provides a rationale for testing nonrenal antifibrotic agents in kidney disease, even though organ-specific modifiers may influence efficacy. The available evidence is still largely preclinical, but it shows that these agents can modulate fibrosis-related pathways such as mechanistic target of rapamycin (mTOR), TGF-β signaling, immune activation, and mechanotransduction (Fig. 4). Pirfenidone and nintedanib, both approved for idiopathic pulmonary fibrosis, provide important precedents. In mouse models of renal injury, pirfenidone suppresses pressure-overload-induced fibrosis and improves renal hemodynamic indices [18], largely through inhibition of TGF-β/Smad signaling and reduction of interstitial fibrosis. Nintedanib, a multitarget tyrosine kinase inhibitor, competitively blocks the ATP-binding pockets of PDGFRs, FGFRs, and vascular endothelial growth factor receptors (VEGFRs). This reduces collagen and fibronectin production by renal fibroblasts, alleviates interstitial fibrosis in UUO and folic acid models, and improves structural remodeling [104]. These data indicate potential value for both agents in kidney fibrosis. Rapamycin, approved for post-transplant indications, suppresses overactive renal mTORC1 signaling and can reduce fibrosis progression through effects on the TGF-β/Smad pathway, lectin signaling, and collagen-related molecules, while also promoting antifibrotic mediators. Persistent mTOR activation is a recognized driver of interstitial fibrosis, and rapamycin-mediated inhibition has been validated in diabetic ischemic renal injury [105]. Among kidney-protective drugs already in use, empagliflozin has emerged as an antifibrotic candidate in diabetic kidney disease. A recently described mechanism involves downregulation of tubular pyruvate kinase muscle isozyme 2 (PKM2): aberrant PKM2 activation in diabetic kidney disease (DKD) promotes metabolic reprogramming and profibrotic factor release, whereas empagliflozin suppresses PKM2 expression and activity in patient samples and animal models [106]. This finding extends the mechanistic landscape of SGLT2 inhibitors and aligns with the broader view that PKM2 dysfunction contributes to CKD progression. Finerenone, a first-line nonsteroidal MRA in DKD, directly blocks excessive MR activation, thereby suppressing downstream inflammatory and TGF-β-dependent fibrotic pathways. It also restores mitochondrial function in diabetic tubules through PI3K/Akt/eNOS signaling, reducing cellular injury and interstitial fibrosis [107]. Among downstream TGF-β-targeted strategies, pamrevlumab (FG-3019), a fully human monoclonal antibody against CCN2/CTGF, inhibits tubular epithelial FAK phosphorylation through the CCN2 module 4-FAK axis and reduces α-SMA-positive myofibroblast accumulation and type I collagen deposition in UUO models [108]. Together, these repurposed agents act on canonical and noncanonical pathways, including TGF-β, mTOR, oxidative stress, and metabolic remodeling. Although most were developed for diabetes, cancer, or other nonrenal diseases, their antifibrotic effects in renal models are increasingly supported by mechanistic and preclinical evidence. This supports drug repurposing in kidney fibrosis, but rigorous evaluation of efficacy and safety within the kidney-specific tissue environment remains essential.

### Emerging cell and receptor targeted therapies

Kidney fibrosis is a defining feature of CKD and an important therapeutic target, but the complex origin and functional heterogeneity of scar-forming cells still hinder clinical intervention. Recent studies identify fibroblasts, pericytes, and myofibroblasts as the principal ECM-producing cells in the kidney and confirm their central role in renal fibrogenesis. These studies also identify PDGFRβ as a potential cell-surface antigen, creating a rationale for antifibrotic chimeric antigen receptor T-cell (CAR-T) therapy. In multiple mouse models of CKD, PDGFRβ-targeted CAR-T cells selectively depleted renal ECM-producing cells and markedly attenuated interstitial fibrosis [109], representing a notable cell-directed strategy for kidney fibrosis. Translation remains challenging, however, because renal ECM-producing cells are functionally heterogeneous, targeting may be incomplete, CAR-T products can cause immunotoxicity and manufacturing costs are high.

Receptor-based strategies directed at the angiotensin II type 1 receptor (AT1R) are also gaining attention because they may reverse kidney fibrosis through more than one mechanism. Losartan, the prototypic AT1R antagonist, exerts antifibrotic effects by downregulating mediators such as TGF-β1, CTGF, PDGF, and Ang II while enhancing interstitial ECM degradation through activation of MMP-2 and MMP-9 [110]. Mechanistically optimized next-generation AT1R-targeted drugs, including the highly selective angiotensin II receptor blocker (ARB) allisartan and the dual AT1R/endothelin A receptor (ETAR) antagonist sparsentan, have entered early clinical evaluation and have shown kidney-targeting and pharmacodynamic advantages over traditional ARBs in phase I studies [111, 112]. Although these agents are currently being tested mainly in CKD and diabetic nephropathy populations with concomitant fibrosis, their ability to suppress fibroblast activation while enhancing interstitial ECM clearance makes them attractive translational candidates for fibrotic kidney disease.

### Antifibrotic therapies in clinical trials

Clinical research in kidney fibrosis has concentrated mainly on a few mechanistic domains: inflammatory suppression, RAAS modulation, metabolic reprogramming, ECM remodeling, and epigenetic regulation. Representative agents include pirfenidone, SGLT2 inhibitors, MRAs, and newer antifibrotic candidates such as TGF-β monoclonal antibodies and integrin αvβ6 inhibitors (Table 2).

**Table 2:** Ongoing or completed clinical trials targeting kidney fibrosis mechanisms and therapies.

Phase	Disease	Trial	Type	*n*	Treatment	Key endpoints	Status	Trial ID
3	CKD (including renal fibrosis)	EMPA-KIDNEY	Interventional	6609	Empagliflozin 10 mg	eGFR; urinary albumin-to-creatinine ratio (UACR); fibrosis biomarkers; composite renal endpoint	Completed	NCT03594110
3	CKD (including renal fibrosis)	DAPA-CKD	Interventional	4304	Dapagliflozin 10 mg sodium-glucose cotransporter 2 inhibitor(SGLT2i) vs placebo	eGFR; UACR; composite renal endpoint; cardiovascular death	Completed	NCT03036150
4	DKD with renal fibrosis	Mechanism of SGLT2 inhibition in diabetic kidney disease	Interventional	20	Canagliflozin 100 mg	Renal MRI-extracellular volume (ECV); urinary TGF-β; eGFR	Recruiting	NCT06291155
3	T2D with CKD (including renal fibrosis)	VERTIS-CV	Interventional	8246	Ertugliflozin 5/15 mg (SGLT2i) vs placebo	eGFR; UACR; composite renal endpoint; cardiovascular death	Completed	NCT01986881
2	Moderate CKD (including renal fibrosis)	Metformin in kidney disease	Interventional	125	Metformin 500–1500 mg vs placebo	leptin–to–adiponectin ratio (LAR); eGFR; vascular health markers (FMD/aPWV)	Completed	NCT02252081
3	T1D + DKD (including renal fibrosis)	SUGARNSALT	Interventional	150	Sotagliflozin (dual SGLT1/2 inhibitor) vs placebo	Annual eGFR decline; UACR; composite renal endpoint	Recruiting	NCT06217302
3	Primary IgA nephropathy (including renal fibrosis)	NefiGard	Interventional	365	Nefecon 16 mg (targeted-release budesonide) vs placebo	2-year time-weighted mean eGFR; urine protein/creatinine; composite renal endpoint	Completed	NCT03643965
3	T2D + DKD with fibrosis	FIDELIO-DKD	Interventional	5734	Finerenone (nonsteroidal MRA) 10/20 mg vs placebo	eGFR; UACR; composite renal endpoint; cardiovascular death	Completed	NCT02540993
3	T2D + DN with fibrosis	SONAR	Interventional	5107	Atrasentan (endothelin receptor antagonist) 0.75 mg QD vs placebo	Composite renal endpoint (eGFR decline ≥50%, ESRD, or renal death)	Terminated	NCT01858532
3	T2D + CKD (including renal fibrosis)	FLOW	Interventional	3533	Semaglutide (GLP-1RA) 0.5/1.0 mg QW vs placebo	eGFR; UACR; composite renal endpoint; cardiovascular death	Completed	NCT03819153
2	CKD with renal fibrosis	TOP-CKD	Interventional	200	Pirfenidone vs placebo	eGFR slope; UACR; composite renal endpoint	Unknown	NCT04258397
2	IgA nephropathy with fibrosis	SC0062 in IgA Nephropathy	Interventional	255	SC0062 (selective endothelin A receptor antagonist) 5/10/20 mg vs placebo	12-week UPCR change; eGFR; safety measures	Active not recruiting	NCT05687890
Observational	Nephrotic syndrome [minimal change disease (MCD)/focal segmental glomerulosclerosis (FSGS)/membranous nephropathy (MN)] with fibrosis	NEPTUNE	Observational	1200	No intervention (prospective follow-up)	Composite renal endpoint; UPCR; pathology fibrosis score; long-term kidney function change	Recruiting	NCT01209000
2/3	Alport syndrome with fibrosis	CARDINAL	Interventional	187	Bardoxolone methyl 100 mg QD vs placebo	48-week eGFR slope; composite renal endpoint; UACR	Completed	NCT03019185
3	ADPKD with renal fibrosis	FALCON	Interventional	667	Bardoxolone methyl vs placebo	eGFR slope, renal functional biomarkers	Terminated	NCT03918447

### SGLT2 inhibitors and metabolic modulators: expanding roles in kidney fibrosis

SGLT2 inhibitors and related metabolic modulators have become a major focus in CKD research because their effects extend beyond glycemic control in diabetic kidney disease and may slow tubulointerstitial fibrotic progression. Their antifibrotic activity is thought to involve reduced oxidative stress in renal tissue, remodeling of tubular energy metabolism, and targeted suppression of TGF-β/Smad signaling. Several clinical trials have assessed these agents in kidney fibrosis [106]. A completed phase 3 study (NCT03594110) evaluated empagliflozin in patients with CKD, including diabetic kidney disease, and supported its ability to modulate fibrosis-related biomarkers, reinforcing its clinical relevance for renal interstitial fibrosis. A phase 3 trial of dapagliflozin (NCT03036150) in CKD with type 2 diabetes showed that the drug could inhibit fibrotic progression, partly by reducing glomerular mesangial matrix expansion; results from this program have been reported in the New England Journal of Medicine. A mechanistic trial of canagliflozin (NCT06291155) in patients with type 2 diabetes and early diabetic kidney disease is currently recruiting and aims to determine how the drug alters intrarenal energy-metabolism transcripts using kidney biopsy and renal magnetic resonance imaging (MRI). The VERTIS-CV study of ertugliflozin (NCT01986881), although designed primarily around cardiovascular safety in type 2 diabetes with cardiovascular disease, has provided indirect renal evidence: subgroup analyses suggest that ertugliflozin lowers proteinuria and slows eGFR decline by suppressing TGF-β/Smad signaling, with favorable effects on fibrosis-related indices. Taken together, these trials support SGLT2 inhibitors as modulators of kidney fibrosis through improved renal energy metabolism, reduced oxidative stress, and suppression of TGF-β/Smad signaling.

Other metabolic modulators are being evaluated in parallel. A completed randomized controlled trial of metformin (NCT02252081) in patients with type 2 diabetes and stage 3–4 CKD showed that adenosine monophosphate-activated protein kinase (AMPK) activation reduced fibrosis-associated markers such as TGF-β1 and collagen III and slowed tubulointerstitial fibrosis. A phase 3 trial of sotagliflozin, a dual SGLT1/SGLT2 inhibitor (NCT06217302), is recruiting patients with type 1 diabetes and diabetic kidney disease to assess whether it can delay renal fibrosis progression through regulation of renal energy metabolism and inhibition of the TGF-β/Smad pathway. These studies support the translational value of metabolic drugs in kidney fibrosis and emphasize the need to convert mechanistic metabolic insights into fibrosis-targeted clinical outcomes. Beyond SGLT2 and dual SGLT1/2 inhibition, the glucagon-like peptide-1 (GLP-1) receptor agonist semaglutide has demonstrated renal protective effects in metabolic kidney disease. The phase 3 FLOW trial (NCT03819153) evaluated once-weekly semaglutide (0.5/1.0 mg) versus placebo in patients with type 2 diabetes and CKD. Results showed a significant reduction in albuminuria and a slower rate of eGFR decline, with exploratory analyses suggesting anti-inflammatory and hemodynamic mechanisms that may indirectly attenuate tubulointerstitial fibrosis. These findings position GLP-1 receptor agonists as complementary metabolic modulators in the antifibrotic landscape of diabetic kidney disease.

### Anti-inflammatory and immunomodulatory agents: targeting inflammation-driven fibrotic remodeling

Inflammation contributes to kidney fibrosis through sustained immune activation, paracrine signaling, and myofibroblast reprogramming. Anti-inflammatory and immunomodulatory strategies are therefore being tested for their capacity to attenuate fibrosis and slow CKD progression. The targeted-release formulation of budesonide (Nefecon) represents a localized anti-inflammatory strategy for immune-complex-mediated kidney fibrosis. The phase 3 NefiGard trial (NCT03643965) evaluated Nefecon 16 mg once daily versus placebo in patients with primary IgA nephropathy and concomitant tubulointerstitial fibrosis. By delivering corticosteroids to the ileocecal region—the presumed site of pathogenic IgA1-producing lymphoid tissue—this approach suppresses mucosal immune activation and downstream renal inflammation. The trial demonstrated a significant reduction in urine protein-to-creatinine ratio and stabilization of eGFR over 2 years, suggesting that localized immunomodulation can slow fibrotic progression in IgA nephropathy. These findings support the principle that upstream suppression of immune-inflammatory drivers can attenuate fibrosis, although direct histological quantification of interstitial collagen reduction remains to be established. Broader application of systemic anti-inflammatory agents in diabetic kidney disease (e.g. the COLICA-DKD colchicine study) has shown reductions in urinary monocyte chemoattractant protein-1 (MCP-1), yet without definitive effects on biopsy-based fibrosis area, underscoring the need for kidney-specific histological endpoints in future anti-inflammatory trials.

### RAAS modulators and mineralocorticoid receptor antagonists: revisiting fibrotic pathways in renal remodeling

RAAS modulators, including MRAs and inhibitors of the angiotensin pathway, have regained attention because their antifibrotic effects may extend beyond conventional hemodynamic control. By suppressing interstitial fibroblast activation, ECM deposition, and tubulointerstitial remodeling, these agents have become core therapies for CKD-associated kidney fibrosis and are now being evaluated directly in fibrosis-focused trials. A phase 3 study (NCT02540993; FIDELIO-DKD) examined finerenone monotherapy in type 2 diabetic kidney disease to determine whether selective MR antagonism can delay interstitial fibrosis, reduce renal function decline, and lower the risk of end-stage renal disease (ESRD). These observations reinforce MRAs as a cornerstone of antifibrotic therapy in diabetic kidney disease, although whether such benefits extend to nondiabetic fibrotic nephropathies requires further investigation. Beyond mineralocorticoid receptor blockade, direct targeting of the renin–angiotensin axis remains central to antifibrotic therapy in CKD. AT1R antagonists not only lower intraglomerular pressure but also suppress downstream TGF-β1, CTGF, and PDGF signaling, thereby reducing interstitial fibroblast activation and enhancing ECM degradation via MMP-2 and MMP-9. In this context, losartan has demonstrated antifibrotic efficacy in experimental models of obstructive and diabetic kidney disease. Mechanistically optimized next-generation agents have now entered clinical evaluation. The dual endothelin type A/AT1R antagonist sparsentan showed superior antiproteinuric efficacy compared with irbesartan in the phase 3 PROTECT trial (NCT03762850) in IgA nephropathy, supporting its potential as a precision antifibrotic agent in immune-complex-mediated glomerular disease. Another novel agent, allisartan, a highly selective AT1R blocker with improved kidney-targeting pharmacokinetics, has shown favorable hemodynamic and antifibrotic profiles in early-phase studies. These developments indicate that refined RAAS modulation—whether through more selective receptor targeting or dual pathway inhibition—continues to offer tangible routes for fibrosis-directed intervention, particularly when combined with SGLT2 inhibitors or nonsteroidal MRAs in multidrug regimens.

### ECM modulators: targeting the structural core of renal remodeling

Abnormal ECM accumulation is a defining pathological event in the progression from CKD to kidney fibrosis. Agents that rebalance ECM synthesis and degradation are therefore generating interest through innovative clinical studies. The TOP-CKD study (NCT04258397) established a noninvasive framework for efficacy assessment by combining quantitative MRI with urinary fibrosis biomarkers, avoiding some limitations of repeated kidney biopsy and enabling broader capture of structural remodeling and functional preservation during ECM-modulating therapy. The phase 3 SONAR trial (NCT01858532) tested atrasentan, a selective endothelin A receptor antagonist, in patients with type 2 diabetes and diabetic nephropathy. By blocking endothelin-1 signaling, atrasentan reduced albuminuria and markers of fibroblast activation; however, the trial was terminated early because of fluid-retention safety signals. These findings highlight both the antifibrotic potential of interrupting endothelin-mediated ECM accumulation and the need for careful management of sodium and water balance when targeting this pathway in CKD. A more targeted clinical study (NCT05687890) in IgA nephropathy examined the effect of a novel endothelin A receptor antagonist on ECM deposition. By blocking endothelin signaling, this agent suppresses fibroblast activation and abnormal accumulation of core ECM components such as collagen. After 24 weeks of intervention, it significantly reduced proteinuria without increasing safety risks such as peripheral edema, offering a precision ECM-targeting approach for immune-complex-mediated renal fibrosis. Because ECM deposition differs across kidney-disease subtypes, these studies illustrate two complementary strategies: broad validation frameworks and subtype-directed precision intervention. They also highlight the translational promise of ECM modulators and the need to optimize dosing according to disease-specific pathology to balance efficacy and safety.

### Genomics and precision-guided trials: anchoring antifibrotic discovery in molecular stratification

Precision-guided clinical trials are changing the study and treatment of kidney fibrosis by linking molecular phenotypes with therapeutic responses. The core idea is to move beyond conventional pathological classification and stratify patients by molecular features, thereby identifying subgroups most likely to benefit from targeted intervention. This approach has already moved into practice. The large observational NEPTUNE cohort (NCT01209000) systematically collected kidney-biopsy tissue from patients with nephrotic syndrome and integrated genomic, transcriptomic, and proteomic analyses to construct molecular subtyping models, including a metabolically unfavorable subtype. This framework showed that molecular signatures of metabolic reprogramming are significantly associated with rapid progression of tubulointerstitial fibrosis, providing both an enrichment strategy and a mechanistic foundation for subsequent antifibrotic trials. In inherited kidney disease with well-defined etiology, precision intervention has already entered the clinic. The CARDINAL phase 2/3 trial in Alport syndrome (NCT03019185) used a randomized, double-blind, placebo-controlled design to evaluate bardoxolone methyl, an Nrf2 activator. The primary endpoint was the change from baseline in eGFR at week 48, and the study demonstrated a significant eGFR improvement with bardoxolone methyl versus placebo. These results provide proof of concept that targeting oxidative stress and mitochondrial dysfunction through Nrf2 activation can modify the course of hereditary fibrotic nephropathy, although long-term clinical outcomes and safety profiles require further evaluation.

### Patient stratification and fibrosis reversibility in clinical design

Clinical trials in kidney fibrosis are increasing, but most still lack standardized precision-enrollment frameworks. Biomarker-defined fibrosis staging and etiologic subtyping have not yet become routine inclusion criteria. Kidney fibrosis arises from diverse causes, including dysregulated glucose metabolism, immune-complex deposition, and urinary tract obstruction, and progresses alongside dynamic interstitial collagen remodeling and tubular injury-repair responses. Earlier identification of patients with potentially reversible fibrosis, together with stratification based on tissue pathology and functional imaging, may improve therapeutic responsiveness. Future studies should integrate genetic susceptibility loci, quantitative imaging markers such as magnetic resonance elastography and ultrasound shear-wave elastography, and cell-specific biomarkers such as fibroblast activation protein, fibronectin, and kidney injury molecule-1. This kind of multidimensional stratification would better match therapeutic targets with the pathological features of fibrosis. It would also reduce trial bias caused by patient heterogeneity and improve the alignment between drug mechanisms and disease biology, thereby strengthening reproducibility and clinical benefit.

## CURRENT LIMITATIONS OF ANTIFIBROTIC THERAPY

Despite extensive preclinical data, major obstacles still limit the clinical translation of antifibrotic drugs in kidney disease. This laboratory-to-clinic gap is evident across several trials. Pirfenidone studies in ADPKD provide one example: although the drug reduces renal collagen deposition and downregulates fibrosis-associated genes such as Col3a1 through inhibition of TGF-β/mTOR signaling, it has produced only modest improvement in blood urea nitrogen and no substantial gain in clinically meaningful renal outcomes. This illustrates a core limitation of broad antifibrotic strategies: biochemical improvement does not necessarily translate into better clinical endpoints [113]. Tolvaptan in ADPKD offers a similar lesson. Although it can modestly slow kidney volume expansion, six-year follow-up has highlighted variable efficacy related to genotype and disease stage, limited benefit in patients with rapid functional decline, and hepatotoxicity-related safety concerns that lead some patients to discontinue treatment [114]. These results point to insufficient target specificity and to deeper systemic bottlenecks. Kidney-specific biomarkers that accurately predict long-term functional decline remain limited, forcing many trials to rely on incompletely validated surrogate endpoints. Systemic drug delivery often fails to achieve adequate penetration and retention within fibrotic interstitial lesions. The redundancy of fibrotic signaling pathways and pronounced patient heterogeneity also continue to weaken single-target approaches. These challenges are changing research strategy. The success of newer agents such as dapagliflozin in CKD may reflect less a strong direct antifibrotic action than an ability to improve renal hemodynamics and slow eGFR decline, thereby indirectly delaying fibrosis through baseline organ protection [115]. This observation suggests that future antifibrotic strategies need to be more integrative. These systemic bottlenecks highlight the urgent need for validated kidney-specific biomarkers, improved drug delivery to fibrotic lesions, and mechanism-based combination approaches that address pathway redundancy and patient heterogeneity.

## SUMMARY AND PROSPECT

Kidney fibrosis is a core pathological process in the progression of many forms of CKD to ESKD and has become a major frontier in nephrology research. This review summarizes the molecular mechanisms governing kidney fibrosis from cellular regulation to tissue remodeling, the use of *in vitro* cell systems, *in vivo* animal models, and organoid models in antifibrotic drug development, and the progress and challenges of candidate agents now in clinical development. Current evidence indicates that sustained renal fibroblast activation is a central event in fibrogenesis, while immune-cell infiltration, mechanical stress, and metabolic dysregulation add further complexity. These translational challenges described above are consistent with the limitations summarized in section ‘CURRENT LIMITATIONS OF ANTIFIBROTIC THERAPY’.

Closing the gap between basic discovery and clinical translation will require multidisciplinary integration and coordinated innovation. The role of mechanotransduction in kidney fibrosis remains incompletely defined, and biomechanically guided interventions need to move from *in vitro* mechanistic studies into standardized clinical evaluation. Interactions between immune cells and kidney-resident cells, particularly macrophage–fibroblast crosstalk, remain an important source of disease-specific targets; single-cell sequencing together with spatial transcriptomics will continue to improve the resolution at which fibrotic heterogeneity can be understood. The integration of noninvasive assessment technologies with AI offers a new route toward precision management of kidney fibrosis [116]. By mining multidimensional data from imaging and molecular biomarkers, AI may support dynamic disease monitoring, subtype classification, and prediction of treatment response, improving individualized therapeutic matching. Human iPSC-based kidney organoids also address some limitations of traditional animal models and, under regulatory frameworks such as the FDA Modernization Act, are gradually being incorporated into high-throughput antifibrotic screening programs, laying the foundation for future organoid-based clinical testing [117]. For example, fibroblast-specific reporter systems in iPSC-derived kidney organoids have identified approved metabolic drugs such as metformin as potential antifibrotic candidates, illustrating the value of organoid screening for drug repurposing.

The development of iPSC organoid technology underscores the importance of interdisciplinary work in antifibrotic drug discovery. As nephrology, systems biology and bioengineering continue to converge, therapeutic development is likely to become more efficient, and precision intervention for kidney fibrosis will move increasingly from the laboratory toward the clinic. Important issues remain unresolved, including standardized disease stratification, validation of model fidelity, and adaptation of regulatory policies. Even so, continued methodological advances make targeted and durable antifibrotic therapies increasingly realistic. Future work should focus on these unresolved questions so that translational kidney fibrosis research can advance both mechanistic understanding and clinical practice.

## Data Availability

No new data were generated or analyzed in support of this review.

## References

[1] Tran T, Song CJ, Nguyen T et al. A scalable organoid model of human autosomal dominant polycystic kidney disease for disease mechanism and drug discovery. Cell Stem Cell. 2022;29:1083–101.e7. 10.1016/j.stem.2022.06.005 35803227 PMC11088748

[2] Huang R, Fu P, Ma L. Kidney fibrosis: from mechanisms to therapeutic medicines. Sig Transduct Target Ther. 2023;8:129. 10.1038/s41392-023-01379-7 PMC10023808 36932062

[3] Zhao M, Wang L, Wang M et al. Targeting fibrosis: mechanisms and clinical trials. Sig Transduct Target Ther. 2022;7:206. 10.1038/s41392-022-01070-3 PMC9247101 35773269

[4] Hong Q, Kim H, Cai G-Y et al. Modulation of TGF-β signaling new approaches toward kidney disease and fibrosis therapy. Int J Biol Sci. 2025;21:1649–65. 10.7150/ijbs.101548 39990662 PMC11844295

[5] Wu W, Wang X, Yu X et al. Smad3 Signatures in renal inflammation and fibrosis. Int J Biol Sci. 2022;18:2795–806. 10.7150/ijbs.71595 35541902 PMC9066101

[6] Wang Y, Li Y, Chen Z et al. GSDMD-dependent neutrophil extracellular traps promote macrophage-to-myofibroblast transition and renal fibrosis in obstructive nephropathy. Cell Death Dis. 2022;13:693. 10.1038/s41419-022-05138-4 35941120 PMC9360039

[7] Chen H, You R, Guo J et al. WWP2 regulates renal fibrosis and the metabolic reprogramming of profibrotic myofibroblasts. J Am Soc Nephrol. 2024;35:696–718. 10.1681/ASN.0000000000000328 38502123 PMC11164121

[8] Rieder F, Nagy LE, Maher TM et al. Fibrosis: cross-organ biology and pathways to development of innovative drugs. Nat Rev Drug Discov. 2025;24:543–69. 10.1038/s41573-025-01158-9 40102636 PMC13264708

[9] He S, Xia T, Guo Z et al. Tracing the origin of myofibroblasts in kidney fibrosis. Nat Commun. 2025;17:653. 10.1038/s41467-025-67373-5 41423454 PMC12815922

[10] Luo L, Wang S, Hu Y et al. Precisely regulating M2 subtype macrophages for renal fibrosis resolution. ACS Nano. 2023;17:22508–26. 10.1021/acsnano.3c05998 37948096

[11] Melkonian AL, Cheung MD, Erman EN et al. Single-cell RNA sequencing and spatial transcriptomics reveal unique subpopulations of infiltrating macrophages and dendritic cells following AKI. Am J Physiol Renal Physiol. 2025;328:F907–20. 10.1152/ajprenal.00059.2025 40331777

[12] Aran D, Looney AP, Liu L et al. Reference-based analysis of lung single-cell sequencing reveals a transitional profibrotic macrophage. Nat Immunol. 2019;20:163–72. 10.1038/s41590-018-0276-y 30643263 PMC6340744

[13] Ramachandran P, Dobie R, Wilson-Kanamori JR et al. Resolving the fibrotic niche of human liver cirrhosis at single cell level. Nature. 2019;575:512–8. 10.1038/s41586-019-1631-3 31597160 PMC6876711

[14] Liu D, Sun H, Li K et al. HIF-1α mediates renal fibrosis by regulating metabolic remodeling of renal tubule epithelial cells. Biochem Biophys Res Commun. 2022;618:15–23. 10.1016/j.bbrc.2022.06.008 35714566

[15] Naas S, Schiffer M, Schödel J. Hypoxia and renal fibrosis. Am J Physiol Cell Physiol. 2023;325:C999–C1016. 10.1152/ajpcell.00201.2023 37661918

[16] Zhao X, Li Y, Yu J et al. Role of mitochondria in pathogenesis and therapy of renal fibrosis. Metabolism. 2024;155:155913. 10.1016/j.metabol.2024.155913 38609039

[17] Li X, Chen J, Li J et al. ATGL regulates renal fibrosis by reprogramming lipid metabolism during the transition from AKI to CKD. Mol Ther. 2025;33:805–22. 10.1016/j.ymthe.2024.12.053 39748508 PMC11853023

[18] Bai X, Nie P, Lou Y et al. Pirfenidone is a renal protective drug: mechanisms, signalling pathways, and preclinical evidence. Eur J Pharmacol. 2021;911:174503. 10.1016/j.ejphar.2021.174503 34547247

[19] Maggisano M, Mondini L, Chernovsky M et al. Safety of nintedanib in a patient with chronic pulmonary fibrosis and kidney disease. Pharmaceuticals. 2024;17:1147. 10.3390/ph17091147 39338310 PMC11434627

[20] Hadpech S, Thongboonkerd V. Epithelial-mesenchymal plasticity in kidney fibrosis. Genesis. 2024;62:e23529. 10.1002/dvg.23529 37345818

[21] Mack M, Yanagita M. Origin of myofibroblasts and cellular events triggering fibrosis. Kidney Int. 2015;87:297–307. 10.1038/ki.2014.287 25162398

[22] Nørregaard R, Mutsaers HAM, Frøkiær J et al. Obstructive nephropathy and molecular pathophysiology of renal interstitial fibrosis. Physiol Rev. 2023;103:2847–92. 10.1152/physrev.00027.2022 PMC10642920 37440209

[23] Wang C, Zhang H, Wang J et al. Aristolochic acid I promotes renal tubulointerstitial fibrosis by activating the mannan-binding lectin serine protease 1-complement system. Food Chem Toxicol. 2025;205:115708. 10.1016/j.fct.2025.115708 40846107

[24] Sharfuddin AA, Molitoris BA. Pathophysiology of ischemic acute kidney injury. Nat Rev Nephrol. 2011;7:189–200. 10.1038/nrneph.2011.16 21364518

[25] Bonventre JV, Yang L. Cellular pathophysiology of ischemic acute kidney injury. J Clin Invest. 2011;121:4210–21. 10.1172/JCI45161 22045571 PMC3204829

[26] Yang L, Si P, Kuerban T et al. UHRF1 promotes epithelial-mesenchymal transition mediating renal fibrosis by activating the TGF-β/SMAD signaling pathway. Sci Rep. 2025;15:3346. 10.1038/s41598-025-86496-9 39870702 PMC11772867

[27] Zhang T, Widdop RE, Ricardo SD. Transition from acute kidney injury to chronic kidney disease: mechanisms, models, and biomarkers. Am J Physiol Renal Physiol. 2024;327:F788–805. 10.1152/ajprenal.00184.2024 39298548

[28] Jacobs ME, de Vries DK, Engelse MA et al. Endothelial to mesenchymal transition in kidney fibrosis. Nephrol Dial Transplant. 2024;39:752–60. 10.1093/ndt/gfad238 37968135

[29] Tuttle KR, Agarwal R, Alpers CE et al. Molecular mechanisms and therapeutic targets for diabetic kidney disease. Kidney Int. 2022;102:248–60. 10.1016/j.kint.2022.05.012 35661785

[30] VD D’A, Chagnac A, de Vries APJ et al. Obesity-related glomerulopathy: clinical and pathologic characteristics and pathogenesis. Nat Rev Nephrol. 2016;12:453–71. 27263398 10.1038/nrneph.2016.75

[31] Jha R, Lopez-Trevino S, Kankanamalage HR et al. Diabetes and renal complications: an overview on pathophysiology, biomarkers and therapeutic interventions. Biomedicines. 2024;12:1098. 10.3390/biomedicines12051098 38791060 PMC11118045

[32] Sanajou D, Ghorbani Haghjo A, Argani H et al. AGE-RAGE axis blockade in diabetic nephropathy: current status and future directions. Eur J Pharmacol. 2018;833:158–64. 10.1016/j.ejphar.2018.06.001 29883668

[33] Li Y, Zhao J, Yin Y et al. The role of IL-6 in fibrotic diseases: molecular and cellular mechanisms. Int J Biol Sci. 2022;18:5405–14. 10.7150/ijbs.75876 36147459 PMC9461670

[34] Roccatello D, Lan H-Y, Sciascia S et al. From inflammation to renal fibrosis: a one-way road in autoimmunity?. Autoimmun Rev. 2024;23:103466. 10.1016/j.autrev.2023.103466 37848157

[35] Sciascia S, Cozzi M, Barinotti A et al. Renal fibrosis in lupus nephritis. Int J Mol Sci. 2022;23:14317. 10.3390/ijms232214317 36430794 PMC9699516

[36] Knoppova B, Reily C, Maillard N et al. The origin and activities of IgA1-containing immune complexes in IgA nephropathy. Front Immunol. 2016;7:117. 10.3389/fimmu.2016.00117 27148252 PMC4828451

[37] Vegting Y, Jongejan A, Neele AE et al. Infiltrative classical monocyte-derived and SPP1 lipid-associated macrophages mediate inflammation and fibrosis in ANCA-associated glomerulonephritis. Nephrol Dial Transplant. 2025;40:1416–27. 10.1093/ndt/gfae292 39694817

[38] Yamashita N, Nakai K, Nakata T et al. Cumulative DNA damage by repeated low-dose cisplatin injection promotes the transition of acute to chronic kidney injury in mice. Sci Rep. 2021;11:20920. 10.1038/s41598-021-00392-6 34686727 PMC8536734

[39] Ludwig T, Riethmüller C, Gekle M et al. Nephrotoxicity of platinum complexes is related to basolateral organic cation transport. Kidney Int. 2004;66:196–202. 10.1111/j.1523-1755.2004.00720.x 15200426

[40] Motohashi H, Sakurai Y, Saito H et al. Gene expression levels and immunolocalization of organic ion transporters in the human kidney. J Am Soc Nephrol. 2002;13:866–74. 10.1681/ASN.V134866 11912245

[41] Guo Y-Y, Liang N-N, Zhang X-Yi et al. Mitochondrial GPX4 acetylation is involved in cadmium-induced renal cell ferroptosis. Redox Biol. 2024;73:103179. 10.1016/j.redox.2024.103179 38733909 PMC11103486

[42] Li S, Cao H, Wang B et al. Effects of heavy metal exposure on kidney transplant recipients: mechanisms and clinical implications for graft failure risk. Front Immunol. 2025;16:1718695. 10.3389/fimmu.2025.1718695 41394865 PMC12698427

[43] Zhou JX, Cheng AS, Chen Li et al. CD74 promotes cyst growth and renal fibrosis in autosomal dominant polycystic kidney disease. Cells. 2024;13:489. 10.3390/cells13060489 38534333 PMC10968819

[44] Gregorio VD, Caparali EB, Shojaei A et al. Alport syndrome: clinical spectrum and therapeutic advances. Kidney Medicine. 2023;5:100631. 10.1016/j.xkme.2023.100631 37122389 PMC10131117

[45] Liu Xi, Liu Z, Wang C et al. Kidney tubular epithelial cells control interstitial fibroblast fate by releasing TNFAIP8-encapsulated exosomes. Cell Death Dis. 2023;14:672. 10.1038/s41419-023-06209-w 37828075 PMC10570316

[46] Pawluczyk IZA, Soares MSF, Barratt WA et al. Macrophage interactions with collecting duct epithelial cells are capable of driving tubulointerstitial inflammation and fibrosis in immunoglobulin A nephropathy. Nephrol Dial Transplant. 2020;35:1865–77. 10.1093/ndt/gfaa079 32830258

[47] Hubbi S, Hao S, Epps J et al. Tumour necrosis factor-alpha at the intersection of renal epithelial and immune cell function. J Physiol. 2025;603:2915–36. 10.1113/JP286756 40349332

[48] Jiang W-J, Xu C-T, Du C-L et al. Tubular epithelial cell-to-macrophage communication forms a negative feedback loop via extracellular vesicle transfer to promote renal inflammation and apoptosis in diabetic nephropathy. Theranostics. 2022;12:324–39. 10.7150/thno.63735 34987648 PMC8690920

[49] Zhang Y, Yang Y, Yang F et al. HDAC9-mediated epithelial cell cycle arrest in G2/M contributes to kidney fibrosis in male mice. Nat Commun. 2023;14:3007. 10.1038/s41467-023-38771-4 37230975 PMC10212923

[50] Chen F, Gao Qi, Wei Ai et al. Histone deacetylase 3 aberration inhibits Klotho transcription and promotes renal fibrosis. Cell Death Differ. 2021;28:1001–12. 10.1038/s41418-020-00631-9 33024274 PMC7937860

[51] Guo S, Zhang Qi, Guo Y et al. The role and therapeutic targeting of the CCL2/CCR2 signaling axis in inflammatory and fibrotic diseases. Front Immunol. 2024;15:1497026. 10.3389/fimmu.2024.1497026 39850880 PMC11754255

[52] Zheng X, Wang J, Chen W et al. Single-cell transcriptomic analysis reveals the association of Ccl6+Ccr2+Arg1+ macrophages with renal interstitial fibrosis in AKI. PLoS One. 2025;20:e0332026. 10.1371/journal.pone.0332026 40953007 PMC12435735

[53] The E, Zhai Y, Yao Q et al. Molecular interaction of soluble Klotho with FGF23 in the pathobiology of aortic valve lesions induced by chronic kidney disease. Int J Biol Sci. 2024;20:3412–25. 10.7150/ijbs.92447 38993571 PMC11234222

[54] Lu Y, Xu S, Tang R et al. A potential link between fibroblast growth factor-23 and the progression of AKI to CKD. BMC Nephrol. 2023;24:87. 10.1186/s12882-023-03125-1 37016338 PMC10074805

[55] Wang H, Gao L, Zhao C et al. The role of PI3K/Akt signaling pathway in chronic kidney disease. Int Urol Nephrol. 2024;56:2623–33. 10.1007/s11255-024-03989-8 38498274

[56] Song J, Chen Y, Chen Y et al. Wnt/β-catenin pathway aggravates renal fibrosis by activating PUM2 transcription to repress YME1L-mediated mitochondrial homeostasis. Biochem Genet. 2025;63:1343–60. 10.1007/s10528-024-10756-y/bib> 38564095

[57] Barrera‐Chimal J, Jaisser F, Anders H‐J. The mineralocorticoid receptor in chronic kidney disease. Br J Pharmacol. 2022;179:3152–64. 10.1111/bph.15734 34786690

[58] Zhao X, Kong Y, Liang B et al. Mechanosensitive Piezo1 channels mediate renal fibrosis. JCI Insight. 2022;7:e152330. 10.1172/jci.insight.152330 35230979 PMC9057604

[59] Xu Yi, Huang Y, Cheng X et al. Mechanotransductive receptor Piezo1 as a promising target in the treatment of fibrosis diseases. Front Mol Biosci. 2023;10:1270979. 10.3389/fmolb.2023.1270979 37900917 PMC10602816

[60] Fu Y, Wan P, Zhang J et al. Targeting mechanosensitive Piezo1 alleviated renal fibrosis through p38MAPK-YAP pathway. Front Cell Dev Biol. 2021;9:741060. 10.3389/fcell.2021.741060 34805150 PMC8602364

[61] Landolt L, Spagnoli GC, Hertig A et al. Fibrosis and cancer: shared features and mechanisms suggest common targeted therapeutic approaches. Nephrol Dial Transplant. 2022;37:1024–32. 10.1093/ndt/gfaa301 33280031

[62] Yamamura Y, Sakai N, Iwata Y et al. Myocardin-related transcription factor contributes to renal fibrosis through the regulation of extracellular microenvironment surrounding fibroblasts. FASEB J. 2023;37:e23005. 10.1096/fj.202201870R 37289107

[63] Liu L, Yu H, Zhao H et al. Matrix-transmitted paratensile signaling enables myofibroblast–fibroblast cross talk in fibrosis expansion. Proc Natl Acad Sci USA. 2020;117:10832–8. 10.1073/pnas.1910650117 32358190 PMC7245086

[64] Zhao Q, Shao T, Zhu Y et al. An MRTF-A–ZEB1–IRF9 axis contributes to fibroblast–myofibroblast transition and renal fibrosis. Exp Mol Med. 2023;55:987–98. 10.1038/s12276-023-00990-6 37121967 PMC10238398

[65] Miranda MZ, Lichner Z, Szászi K et al. Basic biology and role in kidney disease. Int J Mol Sci. 2021;22:6040. 10.3390/ijms22116040 34204945 PMC8199744

[66] Gui Y, Li J, Lu Q et al. Yap/Taz mediates mTORC2-stimulated fibroblast activation and kidney fibrosis. J Biol Chem. 2018;293:16364–75. 10.1074/jbc.RA118.004073 30154246 PMC6200934

[67] He X, Ballestas ER, Zeng Li et al. Podocyte YAP and TAZ hyperactivation drives glomerular epithelial proliferative diseases in mice and humans. Sci Transl Med. 2025;17:eadq3852. 10.1126/scitranslmed.adq3852 40560997 PMC13010181

[68] Zhang M, Tatar M, Gong R. Glycogen synthase kinase 3β: a key player in progressive chronic kidney disease. Clin Sci. 2025;139:605–25. 10.1042/CS20245219 PMC12238819 40526103

[69] Phanish MK, Heidebrecht F, Jackson M et al. Targeting alternative splicing of fibronectin in human renal proximal tubule epithelial cells with antisense oligonucleotides to reduce EDA+ fibronectin production and block an autocrine loop that drives renal fibrosis. Exp Cell Res. 2024;442:114186. 10.1016/j.yexcr.2024.114186 39098465

[70] Ni W‐J, Zhou H, Lu H et al. Genetic and pharmacological inhibition of METTL3 alleviates renal fibrosis by reducing EVL m6A modification through an IGF2BP2-dependent mechanism. Clin Transl Med. 2023;13:e1359. 10.1002/ctm2.1359 37537731 PMC10400756

[71] Wang W, Dai R, Cheng M et al. Metabolic reprogramming and renal fibrosis: what role might Chinese medicine play?. Chin Med. 2024;19:148. 10.1186/s13020-024-01004-x 39465434 PMC11514863

[72] Zhao Y, Fan X, Wang Q et al. ROS promote hyper-methylation of NDRG2 promoters in a DNMTS-dependent manner: contributes to the progression of renal fibrosis. Redox Biol. 2023;62:102674. 10.1016/j.redox.2023.102674 36989575 PMC10074964

[73] Li J, Hu G, Liu W et al. Patchouli alcohol against renal fibrosis of spontaneously hypertensive rats via Ras/Raf-1/ERK1/2 signalling pathway. J Pharm Pharmacol. 2023;75:995–1010. 10.1093/jpp/rgad032 37177974

[74] Huang H, Han Y, Zhang Y et al. Deletion of pyruvate carboxylase in tubular epithelial cell promotes renal fibrosis by regulating SQOR/cGAS/STING-mediated glycolysis. Adv Sci. 2025;12:2408753. 10.1002/advs.202408753 PMC11967762 39836535

[75] Yu Y, Niu Y-Y, Zhang Y-Y et al. Urinary long non-coding RNA GAS5 as a noninvasive diagnostic biomarker for renal fibrosis. Ren Fail. 2025;47:2534493. 10.1080/0886022X.2025.2534493 40685500 PMC12278456

[76] Sun J, Jia Y, Chen S et al. Adipose mesenchymal stem cell-derived extracellular vesicles alleviate renal fibrosis by reducing epithelial-mesenchymal transition via the FOXS1/Wnt/β-catenin signaling pathway. Int Immunopharmacol. 2025;146:113880. 10.1016/j.intimp.2024.113880 39709913

[77] Zhou Y, Ma X-Yu, Han J-Yu et al. Metformin regulates inflammation and fibrosis in diabetic kidney disease through TNC/TLR4/NF-κB/miR-155-5p inflammatory loop. World J Diabetes. 2021;12:19–46. 10.4239/wjd.v12.i1.19 33520106 PMC7807255

[78] Li L, Xiang T, Guo J et al. Inhibition of ACSS2-mediated histone crotonylation alleviates kidney fibrosis via IL-1β-dependent macrophage activation and tubular cell senescence. Nat Commun. 2024;15:3200. 10.1038/s41467-024-47315-3 38615014 PMC11016098

[79] Song J, Chen Y, Chen Y et al. DKK3 promotes renal fibrosis by increasing MFF-mediated mitochondrial dysfunction in Wnt/β-catenin pathway-dependent manner. Ren Fail. 2024;46:2343817. 10.1080/0886022X.2024.2343817 38682264 PMC11060011

[80] Qi R, Wang J, Jiang Y et al. Snai1-induced partial epithelial–mesenchymal transition orchestrates p53–p21-mediated G2/M arrest in the progression of renal fibrosis via NF-κB-mediated inflammation. Cell Death Dis. 2021;12:44. 10.1038/s41419-020-03322-y 33414422 PMC7790819

[81] Wang Z, Yuan A, Liu C et al. Identification of key antifibrotic targets FPR1, TAS2R5, and LRP2BP of valsartan in diabetic nephropathy: a transcriptomics-driven study integrating machine learning, molecular docking, and dynamics simulations. Int J Biol Macromol. 2025;297:139842. 10.1016/j.ijbiomac.2025.139842 39814306

[82] Zhao W-M, Chu F, Zhu J-X et al. Diosmin attenuates UUO-induced renal ferroptosis and fibrosis by inhibiting the HIF-1α/FABP4 signaling axis. Phytomedicine. 2025;141:156738. 10.1016/j.phymed.2025.156738 40233506

[83] Addario G, Moroni L, Mota C. Kidney fibrosis in vitro and in vivo models: path toward physiologically relevant humanized models. Adv Healthc Mater. 2025;14:2403230. 10.1002/adhm.202403230 39906010 PMC11973949

[84] Liu Z-Y, Lin C-H, Wang H-S et al. End-to-end interstitial fibrosis assessment of kidney biopsies with a machine learning-based model. Nephrol Dial Transplant. 2022;37:2093–101. 10.1093/ndt/gfac143 35512604

[85] Gui Y, Wang Y, Palanza Z et al. Calponin 2 harnesses metabolic reprogramming to determine kidney fibrosis. Mol Metab. 2023;71:101712. 10.1016/j.molmet.2023.101712 36963615 PMC10090436

[86] Möhl N, Bouwens D, Abele J et al. Development of a synthetic 3D platform for compartmentalized kidney in vitro disease modeling. Adv Healthc Mater. 2026;15:e03287. 41131838 10.1002/adhm.202503287 PMC12927530

[87] Moran-Horowich A, Lemos DR. Methods for the study of renal fibrosis in human pluripotent stem cell-derived kidney organoids. Methods Mol Biol. 2021;2299:435–45. 10.1007/978-1-0716-1382-5 10.1007/978-1-0716-1382-5_29 34028759

[88] Addario G, Fernández‐Pérez J, Formica C et al. 3D humanized bioprinted tubulointerstitium model to emulate renal fibrosis in vitro. Adv Healthc Mater. 2024;13:e2400807. 10.1002/adhm.202400807 39152919 PMC11582511

[89] Xu Y, Ren Y, Zou W et al. Neutrophil extracellular traps aggravate uremic cardiomyopathy by inducing myocardial fibroblast pyroptosis. Sci Rep. 2025;15:17509. 10.1038/s41598-025-02383-3 40394287 PMC12092652

[90] Cuevas-Delgado P, Miguel V, Rupérez FJ et al. Impact of renal tubular Cpt1a overexpression on the kidney metabolome in the folic acid-induced fibrosis mouse model. Front Mol Biosci. 2023;10:1161036. 10.3389/fmolb.2023.1161036 37377862 PMC10291237

[91] Zhang F, Fan Li, Zhang H et al. Deficiency of IKKα in macrophages mitigates fibrosis progression in the kidney after renal ischemia-reperfusion injury. J Immunol Res. 2021;2021: 1. 10.1155/2021/5521051 PMC8670970 34917688

[92] Bi L, Huang Y, Li J et al. Pirfenidone attenuates renal tubulointerstitial fibrosis through inhibiting miR-21. Nephron. 2022;146:110–20. 10.1159/000519495 34724669

[93] An P, Li X, Zhao Y et al. Curcumin alleviates renal fibrosis in chronic kidney disease by targeting the circ_0008925-related pathway. Ren Fail. 2025;47:2444393. 10.1080/0886022X.2024.2444393 40038566 PMC11884099

[94] Xia Y, Jiang C, Yang M et al. SB431542 alleviates lupus nephritis by regulating B cells and inhibiting the TLR9/TGFβ1/PDGFB signaling. J Autoimmun. 2022;132:102894. 10.1016/j.jaut.2022.102894 36030617

[95] Han Ya-C, Tang S-Qi, Liu Yu-T et al. AMPK agonist alleviate renal tubulointerstitial fibrosis via activating mitophagy in high fat and streptozotocin induced diabetic mice. Cell Death Dis. 2021;12:925. 10.1038/s41419-021-04184-8 34628484 PMC8502176

[96] VF D’A, Takeshita A, Yasuma T et al. Transforming growth factor β1 overexpression is associated with insulin resistance and rapidly progressive kidney fibrosis under diabetic conditions. Int J Mol Sci. 2022;23:14265. 36430743 10.3390/ijms232214265 PMC9693927

[97] Huang J, Shi L, Yang Y et al. Mesenchymal cell-derived exosomes and miR-29a-3p mitigate renal fibrosis and vascular rarefaction after renal ischemia reperfusion injury. Stem Cell Res Ther. 2025;16:135. 10.1186/s13287-025-04226-4 40075481 PMC11905586

[98] Xiong Ya-B, Huang W-Y, Ling X et al. Mitochondrial calcium uniporter promotes kidney aging in mice through inducing mitochondrial calcium-mediated renal tubular cell senescence. Acta Pharmacol Sin. 2024;45:2149–62. 10.1038/s41401-024-01298-5 38789496 PMC11420221

[99] Lu J, Sun W, Liu B et al. Chk2 modulates Bmi1-deficiency-induced renal aging and fibrosis via oxidative stress, DNA damage, and p53/TGFβ1-induced epithelial-mesenchymal transition. Int J Biol Sci. 2024;20:2008–26. 10.7150/ijbs.93598 38617548 PMC11008269

[100] Zhu B, Liu Y, Qi D et al. Renal interstitial fibrosis is reduced in high-fat diet-induced obese pigs following renal denervation from the intima and adventitia of the renal artery. Kidney Blood Press Res. 2022;47:135–46. 10.1159/000521100 34852339

[101] Furukawa T, May CN, Jufar AH et al. Persistent renal hypoxia and histologic changes at 4 weeks after cardiopulmonary bypass in sheep. Anesthesiology. 2025;142:1047–57. 10.1097/ALN.0000000000005452 40106745 PMC12061379

[102] Huang L, Ni J, Duncan T et al. Development of a unilateral ureteral obstruction model in cynomolgus monkeys. Anim Models Exp Med. 2021;4:359–68. 10.1002/ame2.12185 PMC8690991 34977487

[103] Ren Li-Li, Li X-J, Duan T-T et al. Transforming growth factor-β signaling: from tissue fibrosis to therapeutic opportunities. Chem Biol Interact. 2023;369:110289. 10.1016/j.cbi.2022.110289 36455676

[104] Ruiz-Ortega M, Lamas S, Ortiz A. Antifibrotic agents for the management of CKD: a review. Am J Kidney Dis. 2022;80:251–63. 10.1053/j.ajkd.2021.11.010 34999158

[105] Gui Y, Dai C. mTOR signaling in kidney diseases. Kidney360. 2020;1:1319–27. 10.34067/KID.0003782020 35372878 PMC8815517

[106] Cai X, Cao H, Wang M et al. SGLT2 inhibitor empagliflozin ameliorates tubulointerstitial fibrosis in DKD by downregulating renal tubular PKM2. Cell Mol Life Sci. 2025;82:159. 10.1007/s00018-025-05688-8 40237854 PMC12003256

[107] Yao L, Liang X, Liu Y et al. Non-steroidal mineralocorticoid receptor antagonist finerenone ameliorates mitochondrial dysfunction via PI3K/Akt/eNOS signaling pathway in diabetic tubulopathy. Redox Biol. 2023;68:102946. 10.1016/j.redox.2023.102946 37924663 PMC10661120

[108] Amano H, Inoue T, Kusano T et al. Module 4-deficient CCN2/connective tissue growth factor attenuates the progression of renal fibrosis via suppression of focal adhesion kinase phosphorylation in tubular epithelial cells. Mol Cell Biol. 2023;43:515–30. 10.1080/10985549.2023.2253130 37746701 PMC10569360

[109] Zhao S, Li R, Xia Y et al. Targeting ECM-producing cells with CAR-T therapy alleviates fibrosis in chronic kidney disease. Cell Stem Cell. 2025;32:1390–1402.e9. 10.1016/j.stem.2025.07.014 40848726

[110] Wang H, Liu J, Fang F et al. Losartan ameliorates renal fibrosis by inhibiting tumor necrosis factor signal pathway. Nefrología. 2024;44:139–49. 10.1016/j.nefro.2023.09.001 38697694

[111] Schanz M, Seikrit C, Hohenstein B et al. First real-world evidence of sparsentan efficacy in patients with IgA nephropathy treated with SGLT2 inhibitors. Clin Kidney J. 2024;18:sfae394. 39872637 10.1093/ckj/sfae394 PMC11770278

[112] Nagasawa H, Ueda S, Suzuki H et al. Sparsentan is superior to losartan in the gddY mouse model of IgA nephropathy. Nephrol Dial Transplant. 2024;39: 1494–503. 10.1093/ndt/gfae021 38271614 PMC11361813

[113] Remadevi V, Jamadar A, Varghese MM et al. Pirfenidone treatment attenuates fibrosis in autosomal dominant polycystic kidney disease. bioRxiv, 10.1101/2025.08.25.672225, 29 August 2025, preprint: not peer reviewed.

[114] Yamazaki M, Kawano H, Miyoshi M et al. Long-term effects of tolvaptan in autosomal dominant polycystic kidney disease: predictors of treatment response and safety over 6 years of continuous therapy. Int J Mol Sci. 2024;25:2088. 10.3390/ijms25042088 38396765 PMC10888637

[115] Heerspink HJL, Jongs N, Chertow GM et al. Effect of dapagliflozin on the rate of decline in kidney function in patients with chronic kidney disease with and without type 2 diabetes: a prespecified analysis from the DAPA-CKD trial. The Lancet. Lancet Diabetes Endocrinol. 2021;9:743–54. 10.1016/S2213-8587(21)00242-4 34619108

[116] Tang Y, Ke Y, Ma H. Non-invasive assessment techniques for renal fibrosis: advances and perspectives. Ren Fail. 2025;47:2555685. 10.1080/0886022X.2025.2555685 41077637 PMC12517420

[117] Suzuki T, Susa K, Kikuchi H et al. iPSC-based drug discovery identified the Hippo signaling pathway as a therapeutic target in the fibrosis of NPHP1-deficient nephronophthisis. Stem Cell Res Ther. 2025;16:489. 10.1186/s13287-025-04567-0 40968381 PMC12447615

